# Subnanometer Interfacial Hydrodynamics: Spatially Resolved Viscosity and Surface Friction

**DOI:** 10.1021/acs.nanolett.5c03950

**Published:** 2025-10-03

**Authors:** Shane R. Carlson, Roland R. Netz

**Affiliations:** Fachbereich Physik, 9166 Freie Universität Berlin, Arnimallee 14, 14195 Berlin, Germany

**Keywords:** nanofluidics, interfaces, friction, viscosity, soft matter, molecular dynamics simulations

## Abstract

For an accurate description of nanofluidic systems, it is crucial to account for the transport properties of liquids at surfaces on subnanometer scales, where the finite range of surface–liquid interactions implies both spatially extended surface–liquid friction and modified interfacial viscosity. This is accounted for via generalized, position-dependent friction-coefficient and interfacial viscosity profiles, which enable the accurate description of interfacial flow at the nanoscale using the Stokes equation. Such profiles are extracted from nonequilibrium molecular dynamics simulations of water on polar, nonpolar, fluorinated, and unfluorinated alkane and alcohol self-assembled monolayers spanning a wide range of wetting characteristics. The Navier friction coefficient, interfacial viscosity excess, and depletion length are found to be interrelated through power laws and to scale exponentially with the work of adhesion. Our framework establishes a foundation for describing subnanometer interfacial fluid flow with implications for electrokinetics, biophysics, and nanofluidics.

## Graphical abstract

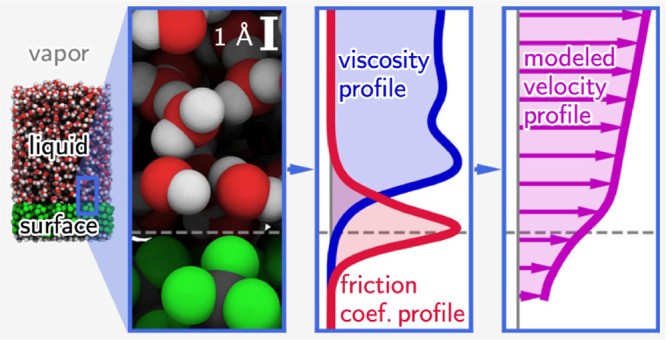

The nanoscale flow of liquids at surfaces, especially water, is of crucial importance in electrokinetics,^1–4^ as well as cell biology and biophysics,^5,6^ such as for transport in transmembrane pores,^7^ bacterial motility,^8^ and hydration layers around biomolecules.^9^ It is the cornerstone of nanofluidics, which has recently attracted a great deal of interest,^10^ with direct applications including carbon nanotube technologies,^11–14^ fabricated nanopores and nanoslits,^15,16^ clean energy,^17,18^ nanofiltration,^19,20^ and biological/medical applications,^21^ such as the manipulation of DNA,^22^ intracellular delivery of biomolecules,^23^ and the analysis of cells, vesicles, and viruses.^24^ We seek to understand, down to the subnanometer scale, how liquids flow adjacent to surfaces, for which the relevant transport properties are the viscosity and surface–liquid friction. As one moves to the subnanometer scale, surface-to-volume ratios become very large, which means that interfacial properties (e.g., surface–liquid friction) become as important as bulk properties (e.g., viscosity).^25,26^ The surface–liquid friction is traditionally characterized for steady-state flow by the Navier friction coefficient, λ, defined via

1 $$F_{f}=-\lambda u_{slip}$$

where *F*_f_ is the surface–liquid friction stress and *u*_slip_ the slip velocity of the liquid, i.e., the relative velocity of the liquid directly adjacent to the surface.^27^ Alternatively, the slip may be cast in terms of slip length *b*, which satisfies the equation λ = η/*b*, where η is the shear viscosity of the liquid.^28^ In the macroscopic context, the slip length is useful for describing low-friction interfaces, such as gases,^29^ or very-high-viscosity liquids, such as polymer melts,^30^ at solid surfaces. For liquids like water, slip lengths are typically on the order of nanometers, and the effect of slippage on flow increases with *b*/*h*, the ratio of slip length to channel size;^25^ therefore, slip lengths for macroscopic flow are vanishingly insignificant, and a no-slip condition is sufficient.^31^ Despite this, finite slippage at solid–liquid interfaces was measured as early as the 1950s in glass capillary channels.^32,33^ More recently, liquids have been shown to flow through carbon nanotubes at rates multiple orders of magnitude larger than would a no-slip flow.^11–14^ Note that eq 1 describes the linear-response regime, i.e., where the slip velocity is low; when it becomes large enough, the relationship is nonlinear.^34,35^

The Navier friction coefficient λ and/or slip length *b* can be extracted from nonequilibrium molecular dynamics (NEMD) simulations where shear is induced by applying external driving forces.^34,36–39^ A typical approach is to induce a steady-state Couette or Poiseuille flow and linearly extrapolate the tangential liquid velocity profile *u*(*z*) past the interfacial position *z*_int_, where *z* is the position normal to the surface, to determine the slip length *b* via

2 $$u_{slip}=u\left(z_{int}\right)=b{\frac{du\left(z\right)}{dz}|}_{z=z_{int}}$$

with *z*_int_ defined, for example, as the (fixed) position of the solid surface^37^ or as the Gibbs dividing surface of the liquid.^39,40^ While this method works reasonably well for low-friction interfaces, problems arise for high-friction interfaces where *u*_slip_ and *b* are small, difficult to measure, and sensitively dependent on the definition of the interfacial position, *z*_int_.

In another approach, equilibrium molecular dynamics (MD) simulations of a solid surface with an adsorbed liquid are carried out, the friction coefficient of the center-of-mass coordinate of the liquid or surface is extracted using Green–Kubo relations, and the Navier friction coefficient is found using the Stokes equation, where a position- and time-independent viscosity η is assumed.^41,42^

These models crucially ignore the nonlocality of intermolecular interactions, which has two key consequences for interfacial hydrodynamics: (1) surface–liquid friction acts over a finite range of distances, as opposed to acting just at a specific position *z*_int_, and (2) the structure and behavior of liquid near an interface are modified, which means that shear viscosity near interfaces is in general dependent on position.

Indeed, viscosity near interfaces has been found to be enhanced when interactions of the liquid with the adjacent phase are strong and reduced when they are weak.^43–45^ Modified interfacial viscosity is of particular importance for nanoconfined liquids.^46,47^ For tangential flow in the *x*-direction, Newton’s law of viscosity gives

3 $$\eta \left(z\right)=\frac{\sigma_{xz}^{ll}\left(z\right)}{\partial_{z}u\left(z\right)}$$

where *∂*_*z*_ *u*(*z*) is the rate of shear and $\sigma_{xz}^{ll}\left(z\right)$ is the liquid–liquid component of the stress opposing the shear.^48^ It is often simpler to calculate an effective viscosity from the total stress via

4 $$\eta_{e\mathrm{ff}}\left(z\right)=\frac{\sigma_{xz}^{ll}\left(z\right)+\sigma_{xz}^{sl}\left(z\right)}{\partial_{z}u\left(z\right)}$$

where $\sigma_{xz}^{sl}\left(z\right)$ is the surface–liquid stress, e.g., for a liquid between two oppositely moving surfaces,^40,43,49^ gravity-driven flow,^50–54^ or flow driven by electric fields^55^ or momentum exchange.^56^ Near the interface, however, η_eff_(*z*) differs from the true viscosity η­(*z*) due to the sizable contribution of surface–liquid friction.^57,58^ Thus, to accurately describe interfacial hydrodynamics at the nanoscale, we separately take into account the position dependence of the surface–liquid friction and interfacial viscosity, extracting both from simulations.

To this end, we carry out steady-state driven-flow NEMD simulations of liquid water slabs on self-assembled monolayer (SAM) surfaces with widely varying wetting and friction properties. Above each water slab is a vacuum in which a water vapor phase forms. This open system geometry circumvents the strong sensitivity of viscosity to nanochannel width arising from the incommensurability of discrete liquid molecule layers.^59,60^ The SAMs comprise decane (H-SAM), decane with the top eight carbons perfluorinated (F-SAM), or decanol (α-SAM), where the partial charges on the OH groups are scaled by a factor α ∈ {0, 0.5, 0.6, 0.7, 0.8, 0.9, 1}. The systems are illustrated in Figure 1a, although the production simulation systems are larger than those shown.

**Figure 1:**
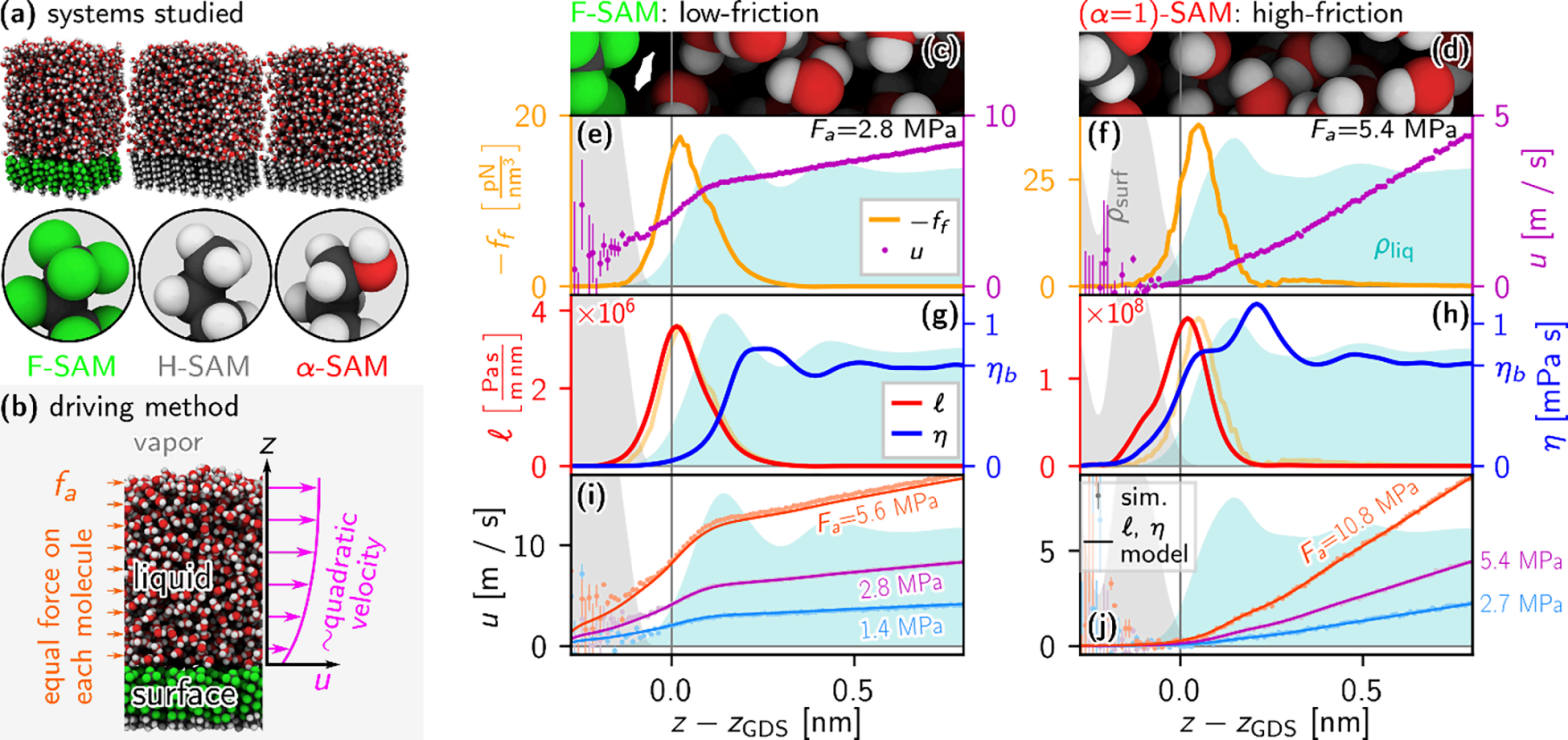
(a) Snapshots of the SAM surfaces studied, along with close-ups of the SAM molecule headgroups. (b) Schematic illustrating the NEMD method used. The SAM is restrained at the bottom, while a constant force acts equally on each liquid molecule. (c–j) Friction-coefficient and viscosity profile extraction for the low-friction F-SAM/water system (left column) and high-friction (α = 1)-SAM/water system (right column). The mass densities of the surface (ρ_surf_) and liquid (ρ_liq_) are plotted as shaded areas with arbitrary *y*-scaling in all plots. Plots are over *z* relative to the water Gibbs dividing surface position *z*_GDS_. (c and d) Simulation snapshots for each respective system. (e and  f) Plots of extracted surface–liquid friction force, *f*_f_(*z*), and liquid velocity, *u*(*z*), profiles. The applied driving stress *F*_a_ (see eq 10) is printed in each plot. (g and  h) Plots of friction-coefficient profiles l ­(*z*) and viscosity profiles η­(*z*), which are calculated from *f*_f_(*z*) and *u*(*z*) profiles (see eqs 6 and 13, respectively). Force profiles *f*_f_(*z*) are also plotted schematically as translucent orange curves for comparison to l ­(*z*). Driven-flow simulations with larger driving stresses are used to calculate η­(*z*): *F*_a_ = 11.2 MPa for the F-SAM, and *F*_a_ = 21.6 MPa for the (α = 1)-SAM. (i and  j) Velocity profiles *u*(*z*) extracted from driven-flow simulations (points with error bars), compared with those calculated using l ­(*z*) and η­(*z*) from the row above by solving eq 14 numerically (solid lines).

The simulations are carried out in GPU-enabled single-precision GROMACS 2023.3 ^61,62^ using the leapfrog integrator^63^ with a 2 fs time step. The velocity-rescaling (CSVR) thermostat is applied to all atoms with a target temperature of 300 K.^64^ Because longer force cutoffs are superior for modeling interfacial properties, the Lennard-Jones forces are treated using force switching between 1.9 and 2 nm.^65,66^ Electrostatic forces are modeled using particle-mesh Ewald beyond 2 nm.^67^ SAM molecules are modeled using the OPLS all-atom (OPLS-AA) force field^68–70^ with selected dihedrals optimized.^65^ Water is modeled using the SPC/E water model.^71^ Further simulation details can be found in section S1 of the Supporting Information.

The water is driven along the *x*-direction, tangential to the SAM, by a constant gravity-like force acting on all liquid atoms; i.e., the force on each atom is proportional to its mass. In the bulk, where the liquid density is constant, this results in a constant force density and a quadratic flow profile with the vertex of the parabola at the liquid–vapor interface, i.e., a half-Poiseuille flow, as illustrated in Figure 1b.

Panels c–j of Figure 1 illustrate the workflow for extraction of friction-coefficient and interfacial viscosity profiles from the driven-flow simulations for the low-friction, fluorinated F-SAM (left column) and the high-friction, polar (α = 1)-SAM (right column). Panels c and d of Figure 1 show snapshots of the simulated systems, zoomed in on the respective interfacial regions of interest. Panels e and f of Figure 1 plot extracted profiles *u*(*z*), the liquid velocity, and *f*_f_(*z*), the friction force per unit volume on the centers of mass of liquid molecules due just to the surface (and not to adjacent liquid), as a function of the *z*-position of liquid molecule centers of mass. The slip at the low-friction, hydrophobic F-SAM is apparent in *u*(*z*); i.e., *u*(*z*) is clearly positive even approaching the surface. For the (α = 1)-SAM on the other hand, slip is not apparent, and *u*(*z*) is convex near the interface, making the definition of a slip velocity or slip length difficult. It is evident in panels e and f of Figure 1 that *f*_f_(*z*) is not confined to a single position *z*_int_ but instead acts on the liquid over several angstroms along the *z*-direction for both surfaces. This illustrates why the surface–liquid friction force should not be ignored in viscosity profile calculations.

The total surface–liquid friction stress *F*_f_ is given by

5 $$F_{f}=\int dz\;f_{f}\left(z\right)$$

In the linear-friction regime, a local, position-dependent friction coefficient l ­(*z*) is defined via

6 $$f_{f}\left(z\right)=-l\left(z\right)u\left(z\right)$$

Combining eqs 1, 5, and 6 yields

$$\lambda u_{slip}=\int dz\;l\left(z\right)u\left(z\right)$$ 7

Equation 7 holds for arbitrary profiles *u*(*z*) in the linear regime, including constant *u*(*z*), with *u* = *u*_slip_, from which follows an expression for the Navier friction coefficient in terms of the microscopically defined friction-coefficient profile

8 λ=∫dzl(z)

which, crucially, does not rely on the definition of an interfacial position *z*_int_. Equations 7 and 8 can be combined to give the corresponding slip velocity

9 $$u_{slip}=\frac{\int dz\;l\left(z\right)u\left(z\right)}{\int dz\;l\left(z\right)}$$

which takes the form of a weighted mean over *u*(*z*). Thus, from eq 6, it follows that the Navier friction law, eq 1, holds for λ and *u*_slip_ as defined in eqs 8 and 9 (see section S2 for a more complete derivation). Friction-coefficient profiles l ­(*z*), calculated via eq 6, are plotted for the F-SAM/water and (α = 1)-SAM/water systems in panels g and h, respectively, of Figure 1. Forces *f*_*f*_(*z*) are also reproduced in panels g and h of Figure 1 as translucent orange curves with an arbitrary scaling factor for comparison to l ­(*z*). As one might expect, the friction-coefficient profile for the very hydrophilic (α = 1)-SAM is (two) orders of magnitude higher than that of the hydrophobic F-SAM.

Turning from the surface–liquid friction to the interfacial viscosity, we begin by denoting the driving force density applied to the liquid in the *x*-direction, *f*_a_(*z*). Though *f*_a_(*z*) can take any form, for the gravity-driven flow studied in this work

10 $$f_{a}\left(z\right)=\frac{F_{a}\;\rho_{liq}\left(z\right)}{\int dz^{\prime }\;\rho_{liq}\left(z^{\prime }\right)}$$

where ρ_liq_(*z*) is the liquid density profile and *F*_a_ is the total applied driving stress. The total external force density on the liquid, *f*(*z*), can be decomposed as

11 $$f\left(z\right)=f_{a}\left(z\right)+f_{f}\left(z\right)$$

For tangential flow on a planar surface, fluid motion is described by the linear Stokes equation^27,72,73^

12 $$f\left(z\right)=-\partial_{z}\left[\eta \left(z\right)\partial_{z}u\left(z\right)\right]$$

where η­(*z*) is a local, position-dependent shear viscosity. Nonlocal viscosity effects must be accounted for only when the rate of shear changes significantly over the intermolecular interaction range.^74^ See section S3 for derivations of the Navier–Stokes and Stokes equations and related discussion. Letting *z* = *z*_0_ denote a position below the liquid phase, eq 12 can be integrated to give

13 $$\eta \left(z\right)=-\frac{1}{\partial_{z}u\left(z\right)}\int_{z_{0}}^{z}dz^{\prime }\;f\left(z^{\prime }\right)$$

which is equivalent to eq 3, i.e., liquid–liquid stress $\sigma_{xz}^{ll}\left(z\right)$ balances total external stress. A more thorough derivation and discussion can be found in section S4. Viscosity profiles η­(*z*), calculated via eq 13, are plotted for the F-SAM/water and α-SAM/water systems in panels g and h, respectively, of Figure 1 (see sections S5 and S6 for further details and plots of the extraction of l ­(*z*) and η­(*z*)).

In order to model Stokes flow, eqs 6 and 11 are substituted into the Stokes equation (eq 12), which yields

14 $$l\left(z\right)u\left(z\right)=f_{a}\left(z\right)+\partial_{z}\left[\eta \left(z\right)\partial_{z}u\left(z\right)\right]$$

Having extracted l ­(*z*) and η­(*z*) for a specific surface–liquid interface at a given temperature and pressure, *u*(*z*) may be found by solving eq 14 numerically for an arbitrarily chosen applied force profile *f*_a_(*z*) (see section S7). Panels i and j of Figure 1 compare velocity profiles extracted from NEMD simulations with those calculated by solving eq 14. It is assumed that *∂*_*z*_ *u*(*z*) = 0 at the liquid–vapor boundary and *u*(*z*) = 0 at the surface. The overall modeling of the flow is very accurate, which validates our model, including the locality of l ­(*z*) and η­(*z*), and indicates that the system is in the linear-response regime for both viscosity and friction (the linear response is also verified in section S8). The same comparison between the l –η model and NEMD simulation data is carried out for all systems studied in this work in section S9. In section S10, we show that failing to account for the position dependence of either l ­(*z*) or η­(*z*) leads to models that fail to accurately capture the flow behavior near the interface.

The friction-coefficient and viscosity profiles for all systems are plotted in Figure 2. Wetting is a natural way to classify surface–liquid interactions, so we extract water contact angles of all systems from equilibrium MD simulations of cylindrical droplets^66^ and extrapolate the droplet size to the macroscopic limit.^65,75–77^ The contact angle extraction is detailed in section S11. Figure 2a shows a simulation snapshot of such a cylindrical water droplet on an F-SAM, with the contact angle indicated schematically, and the resulting contact angles for all systems, which range from the very hydrophobic F-SAM (θ = 125°) to the fully wetted (α = 1)-SAM.

**Figure 2:**
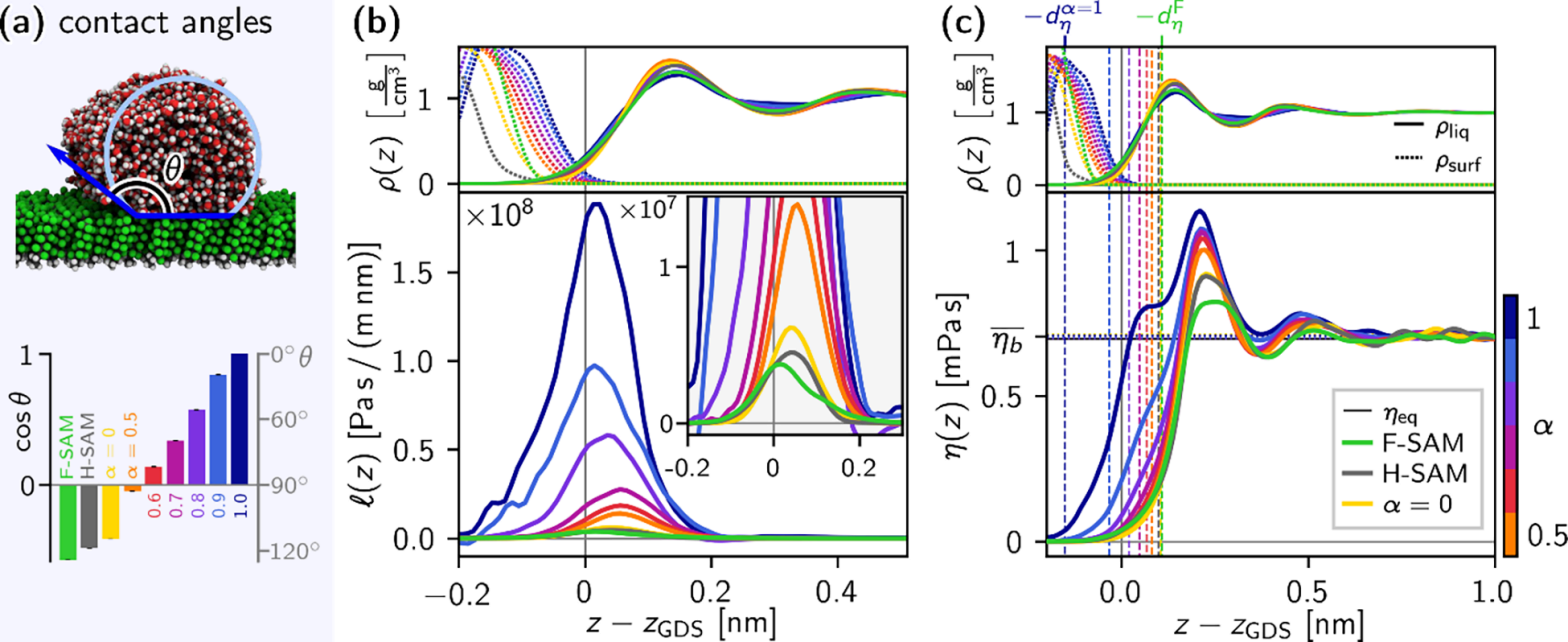
Comparison of friction-coefficient profiles and viscosity profiles of water on different SAM surfaces, plotted over *z* relative to *z*_GDS_. As a positional reference, the mass densities of the surface, ρ_surf_(*z*), and liquid, ρ_liq_(*z*), for all systems are plotted in the top panels as dotted and solid lines, respectively. (a) Water contact angles θ, from droplet simulations for all surfaces. Also shown is a schematic illustrating the contact angle θ, overlaid on a simulation snapshot of a cylindrical water droplet on an F-SAM. The (α = 1)-SAM was fully wetted by water, so we show it as having a contact angle of 0°. (b) Friction-coefficient profiles l ­(*z*) calculated via eq 6. The inset shows the same data zoomed in on a smaller range of *y* values. (c) Viscosity profiles η­(*z*) calculated via eq 13. The average of each viscosity profile in the bulk, η_b_, is shown as a horizontal dotted line. $\overset{®}{\eta_{b}\;}=0.706$ mPa s; cf. η_eq_ = 0.698 mPa s, from an equilibrium bulk water simulation (solid black line). Viscosity dividing surfaces *z*_η_ from each viscosity profile are shown as vertical dashed lines.

Figure 2b shows friction-coefficient profiles l ­(*z*), with the corresponding mass-density profiles shown above as a positional reference. As the friction coefficient varies by more than an order of magnitude, the curves for the low-friction surfaces are difficult to discern in the main plot, and the same data are plotted over a smaller range of *y* values in the inset. The friction-coefficient profiles extend over several angstroms, with full width at half-maximum values ranging from 1.1 to 1.8 Å across all systems. This reveals that at subnanometer scales, friction should not be treated as acting only at a single position *z*_int_.

The viscosity profiles η­(*z*) are shown in Figure 2c. Note that these all decay to zero in the region of the SAM, which has been observed before for Lennard-Jones fluids.^48^ If surface friction is ignored, as in eq 4, the resulting effective viscosity will typically be found to diverge near the interface (see section S12 for details). The viscosity profiles for all systems converge very near the same value in the bulk, as expected. For each system, the viscosity value in the bulk, η_b_, is calculated as the mean of η­(*z*) between 1.5 and 2.5 nm from the top carbon atoms. These are shown as dotted horizontal lines, and they agree well, all falling within the range ${\mathrm{\eta ̅}}_{b}\pm 0.007$ mPa s, where ${\mathrm{\eta ̅}}_{b}=0.706$ mPa s is their mean value, which itself agrees well with η_eq_ = 0.698 mPa s, calculated via the Green–Kubo relation from an equilibrium MD simulation where the same water force field is used (see section S13 for details).^78–81^

Alongside the viscosity profiles η­(*z*) in Figure 2c, viscosity dividing surfaces *z*_η_ (where the interfacial viscosity excess vanishes) are shown as vertical dashed lines and differ from the Gibbs dividing surface *z*_GDS_ by up to about 1 Å in either direction. This reveals that *z*_GDS_ is not the relevant interfacial position for liquid viscosity and highlights the importance of treating the position dependence of the interfacial viscosity at subnanometer scales. We define the interfacial viscosity excess distance as *d*_η_ = *z*_GDS_ – *z*_η_. Thus, a positive *d*_η_ indicates a viscosity excess at the interface, while a negative *d*_η_ indicates a deficit. The viscosity excess in Figure 2c increases significantly as the surfaces become more hydrophilic, with a shoulder forming in η­(*z*) at the first hydration layer nearest the surface, which likely results from the conformational rigidity of water hydrogen-bonded to, or otherwise strongly interacting with, the surface, preventing other water molecules from easily flowing past. One interesting aspect visible in all of the systems is the apparent disagreement between the viscosity and density profiles. Naively, one might expect the viscosity to increase with density, but we observe the main peak of each viscosity profile to be shifted bulkward relative to that of the density profile. This is likely related to water orientation and especially hydrogen bonding near the interface. Indeed, we show that there is a deficit of water–water hydrogen-bonded molecules near the interface for all surfaces in section S14.

In Figure 3, we explore the relationships among the Navier friction coefficient λ, interfacial viscosity excess distance *d*_η_, wetting coefficient *k* = cos θ, and depletion length δ. The depletion length is the distance between the adjacent Gibbs dividing surfaces of the SAM and water phases, as illustrated in Figure 3a, where the Gibbs dividing surface of a phase is the thermodynamically relevant interfacial position (see section S15). For the SAMs, which are chemically inhomogeneous near the interface, the Gibbs dividing surfaces are calculated as the position where the excess of the packing density ϕ­(*z*) vanishes (see section S16).

**Figure 3:**
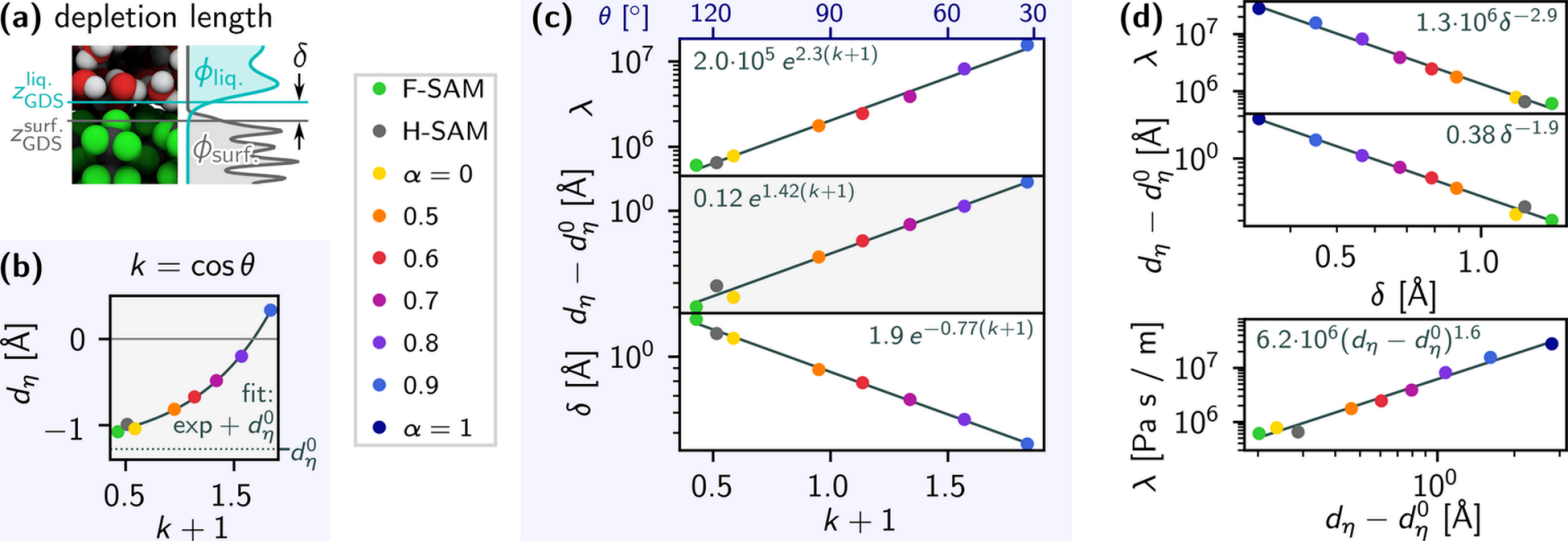
Analysis of relationships among surface–liquid friction, viscosity excess, depletion, and wetting for water on SAMs. (a) Schematic illustrating the depletion length δ, i.e., the distance between the Gibbs dividing surfaces of the liquid and solid. (b) Viscosity excess distance, *d*_η_, as extracted in Figure 2c, over *k* + 1, where *k* = cos θ is the wetting coefficient. The data are fit with an exponential function plus a constant $d_{\eta }^{0}$ , which is subtracted from *d*_η_ in parts c and d. (c) Plots over *k* + 1 of the Navier friction coefficient λ (Pa s m^–1^) extracted by integrating l ­(*z*) in Figure 2b, viscosity excess distance $d_{\eta }-d_{\eta }^{0}$ (see part b), and depletion length δ (see part a), along with exponential fits of the data. The data for the (α = 1)-SAM have been omitted, as *k* is undefined for full wetting. The $d_{\eta }-d_{\eta }^{0}$ data and fit from part b are replotted on a log–linear scale to demonstrate that the data fall on a straight line and agree well with the fit. (d) Relationships among λ, *d*_η_, and δ plotted on a log–log scale, alongside power law fits.

For partial wetting, the wetting coefficient *k* is related to the areal work of adhesion of the surface *W* via the Young–Dupré equation

15 $$W=\gamma_{lv}\left(k+1\right)$$

where γ_lv_ is the liquid–vapor interfacial tension of the liquid. Thus, we plot *k* + 1 rather than θ, and because eq 15 holds only for partial wetting, we do not include the data for the (α = 1)-SAM in plots involving *k* + 1.

In Figure 3b, *d*_η_ is plotted over *k* + 1 alongside a least-squares fit of an exponential function plus a constant, which we call $d_{\eta }^{0}$ . From the fitted function, we calculate *d*_η_(*k* = −1) = −1.25 Å; i.e., there is an interfacial viscosity deficit of 1.25 Å in the dewetting limit, where θ = 180°, which should be characteristic of the water model alone. Figure 3c comprises log–linear plots of λ, $d_{\eta }-d_{\eta }^{0}$ , and δ over *k* + 1, alongside exponential fits. The fits of λ and δ are performed in logarithmic space.

From transition-state theory,^82,83^ it can be shown that $\lambda \propto e^{\beta U_{0}}$ for small driving forces, where *U*_0_ is the barrier height of the corrugated energy landscape seen by a liquid molecule at a surface and $\beta ={\left(k_{B}T\right)}^{-1}$ is the inverse thermal energy. From there, it can be shown under certain approximations that

16 $$\lambda \propto e^{A\left(k+1\right)}$$

where *A* is a positive constant. Indeed, we find λ to be exponential in *k* + 1 in Figure 3c, where the fit gives *A* ≈ 2.3. Previous works have derived other friction–barrier height relationships, including $\lambda \propto e^{\beta U_{0}}/U_{0}$ and $\lambda \propto 1+{\left(\beta U_{0}\right)}^{2}/16$ .^49,84,85^ Fits of these functions to our λ­(*k*) data, a derivation of eq 16, and related discussion can be found in section S17. The middle panel of part c shows the same fit as part b, but shifted by $-d_{\eta }^{0}$ and on a log–linear scale, where the data indeed appear to fall on a straight line, indicating exponential behavior. The bottom panel of part c plots the depletion length, which appears to decay exponentially.

Figure 3d examines the relationships among λ, $d_{\eta }-d_{\eta }^{0}$ , and δ. As these all appear to be exponential in *k* + 1, we expect them to relate to one another via power laws and accordingly plot them on a log–log scale, where the data indeed appear to be linear. The data are fitted by taking the logarithm of both data sets and carrying out a linear fit. These fits yield λ ∝ δ^–2.9^, $d_{\eta }-d_{\eta }^{0}\propto \delta^{-1.9}$ , and $\lambda \propto {\left(d_{\eta }-d_{\eta }^{0}\right)}^{1.6}$ .

We have shown that both inhomogeneous friction-coefficient and interfacial viscosity profiles are important for understanding subnanoscale interfacial flow and that properly decoupling the two allows for the calculation of useful quantities, such as the surface viscosity excess and Navier friction coefficient, without *a priori* recourse to arbitrarily defined interfacial positions. Going forward, these methods for accurately decoupling surface–liquid friction and interfacial viscosity will lend themselves well to the analysis of flow on subnanometer scales for a wide variety of systems, such as systems with liquid mixtures, nanochannels, and charged surfaces and/or electrolyte solutions, which are relevant for electrokinetics. The empirical relations we find among friction coefficient λ, viscosity excess *d*_η_, depletion length δ, and wetting coefficient *k* allow for the parameter-free modeling of interfacial flow at smooth surfaces of arbitrary polarity.

## Supplementary Material



## Notes

Simulation Details (section S1), Position-Dependent Surface–Liquid Friction (section S2), Navier–Stokes and Stokes Equations for Position-Dependent Viscosity (section S3), Calculation of Position-Dependent Viscosity (section S4), Fits of Velocity Profile Tails (section S5), Calculation of Friction-Coefficient and Viscosity Profiles (section S6), Numerically Solving the Stokes Equation (section S7), Verifying Linear-Response Regime by Velocity Profile Fits (section S8), Modeling Flow for All Systems (section S9), Other Approaches to Modeling Flow (section S10), Contact Angles (section S11), Effective Viscosity Profiles (section S12), Bulk Shear Viscosity from the Green–Kubo Relation (section S13), Hydrogen Bonding Near the Interface (section S14), Gibbs Dividing Surface (section S15), Depletion Length (section S16), and The Friction–Wettability Relationship (section S17) (PDF)
