# Supplementary material for: Subnanometer Interfacial Hydrodynamics: Spatially Resolved Viscosity and Surface Friction
Source: Nano Lett. 2025 Oct 3;25(43):15605–12. doi: 10.1021/acs.nanolett.5c03950 (PMC12576822; doi:10.1021/acs.nanolett.5c03950)
Supplement: Supplementary file 1 [file nl5c03950_si_001.pdf]

Supporting Information: Subnanometer Interfacial Hydrodynamics: Spatially Resolved Viscosity and Surface Friction

Shane R. Carlson[1] and Roland R. Netz*[1]

[1]Fachbereich Physik, Freie Universität Berlin, Arnimallee 14, 14195 Berlin, Germany

Contents

S1	Simulation Details	1
S2	Position-Dependent Surface–Liquid Friction	3
S3	Navier–Stokes and Stokes Equations for Position-Dependent Viscosity	3
S4	Calculation of Position-Dependent Viscosity	5
S5	Fits of Velocity Profile Tails	5
S6	Calculation of Friction-Coefficient and Viscosity Profiles	5
S7	Numerically Solving the Stokes Equation	9
S8	Verifying Linear-Response Regime by Velocity Profile Fits	10
S9	Modeling Flow for All Systems	11
S10	Other Approaches to Modeling Flow	12
S11	Contact Angles	13
S12	Effective Viscosity Profiles	14
S13	Bulk Shear Viscosity from the Green–Kubo Relation	15
S14	Hydrogen Bonding Near the Interface	17
S15	Gibbs Dividing Surface	18
S16	Depletion Length	18
S17	The Friction–Wettability Relationship	19

S1 Simulation Details

We carry out nonequilibrium force-field molecular dynamics (MD) simulations of gravity-driven liquid water slabs on alkane, fluoroalkane, and alcohol self-assembled monolayer (SAM) surfaces. These surfaces vary widely in their wetting and friction properties. All system boundaries are periodic, with the surfaces and water slabs contiguous across the boundaries in the xy-plane. The water slabs consist of 8192 water molecules and are roughly 4 nm thick. See Figure S1 for a snapshot of a simulated system. Above each is vacuum in which a water vapor phase may form.

To prevent molecules from crossing the periodic boundary in the z-direction and adhering to the bottom of the SAM, a water-confining potential is applied, which is also illustrated in Figure S1. This potential consists of a flat bottom and harmonic sides and applies only to water molecules and only along the z-direction. The lower harmonic part of the potential is positioned below the SAM, where water molecules do not reach in any case. The entire liquid water slab sits in the flat bottom of the potential, so the potential does not act on the water molecules there. The upper harmonic part is positioned above the top of the water slab, and acts on water molecules in the vapor phase that are above position z_c with force

$$\boldsymbol{F}_c = -k\,(z - z_c)\,\hat{z}\,. \tag{S1}$$

This reflects water molecules downward and back toward the water slab. The harmonic parts have spring constants of $k = 79.997$ kJ/(mol nm^2) for the water oxygens and $k = 5.040$ kJ/(mol nm^2) for the water hydrogens, so that an equal acceleration is applied to both atom species.

The SAMs are comprised of decane (H-SAM), decane with the top eight carbons perfluorinated (F-SAM), or decanol (α-SAM), where the partial charges on the OH group of the heads are scaled by a factor $\alpha \in \{0, 0.5, 0.6, 0.7, 0.8, 0.9, 1\}$, which changes the electric dipole. Snapshots of the molecules are shown in Figure S1. The bottom carbon atoms of the SAM molecules (the ones furthest from the water slab) are restrained to points in space arranged in a hexagonal grid in the xy-plane via a harmonic potential with a spring constant of $k = 25000$ kJ/(mol nm^2). The nearest-neighbor distance of the grid points, i.e., the "grafting distance", is set to 5.9 Å for the F-SAM and 4.97 Å for the H-SAM and α-SAMs. These values are in accordance with experimental findings for F-SAMs and H-SAMs on a gold surface, namely Au(111).[1–6] These SAM grafting distances are discussed at length in Ref. 7. The H-SAM and α-SAMs are 18×16 molecules in a simulation box of $8.946 \times 6.887 \times 40$ nm, while the F-SAM is 14×14 molecules in a simulation box of $8.260 \times 7.153 \times 40$ nm.

The simulations are carried out in GPU-enabled single-precision GROMACS version 2023.3,[8,9] using the leapfrog integrator[10] with a time step of 2 fs. The velocity-rescaling (CSVR) thermostat is applied to all atoms with a target temperature of 300 K and a coupling time-constant `tau-t` of 1 ps.[11] Longer force cutoffs have been shown to be superior for modeling interfacial properties.[7,12] Therefore, the Lennard–Jones forces are modeled using a force-switching scheme between 1.9 and 2 nm. Electrostatic forces are modeled using the particle-mesh Ewald (PME) method beyond the real-space cutoff of 2 nm.[13]

*rnetz@physik.fu-berlin.com

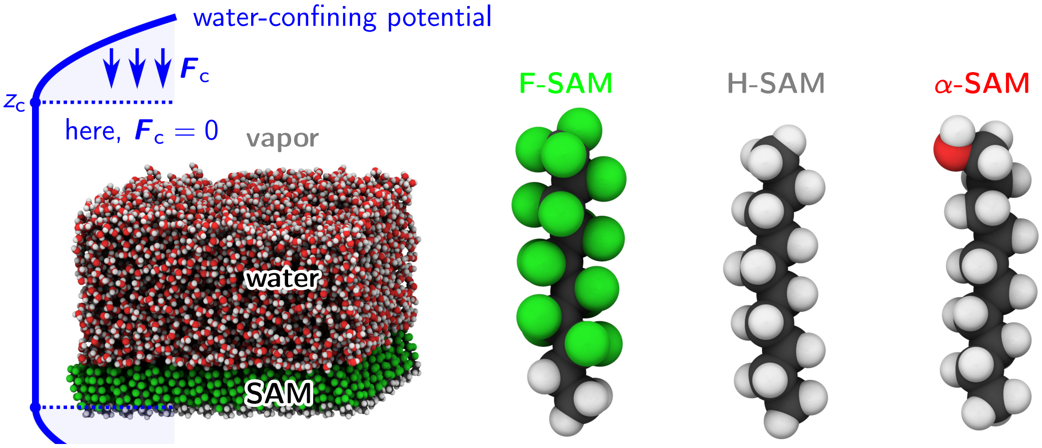

Figure S1: **Left:** A snapshot of the production simulation system for the F-SAM, which consists of a close-packed 14 × 14 grid of molecules with an 8192-molecule water slab. Also shown here schematically is the water-confining potential, which acts with a harmonic force only on water molecules above z_c. **Right:** Snapshots of single SAM molecules for the F-SAM, H-SAM, and an α-SAM. Dark gray atoms are C, light gray H, red O, and green F. Partial charges are tuned on the OH-groups of the α-SAM.

A force and potential correction for the electrostatic PME is used to account for interactions between system replicas along the z-direction by setting `ewald-geometry = 3dc`. SAM molecules are modeled using the OPLS All-Atom (OPLS-AA) force field[14–16] with selected dihedrals optimized for the F-SAM.[7] All covalent bond lengths involving hydrogen atoms are fixed by setting `constraints = hbonds`. Water is modeled using the SPC/E water model.[17] SPC/E was originally optimized with a 0.9-nm Lennard–Jones cutoff, and, because a longer cutoff is employed in this work, the liquid properties can be expected to differ. Table S1 compares the properties of SPC/E water at 300 K for these two Lennard–Jones cutoff schemes: the water density and shear viscosity of the bulk liquid are virtually unchanged, but as the cutoff increases, the surface tension increases significantly, toward the experimental value of 71.99(36) mJ/m^2 at 25° C.[18]

	0.9 nm potential-shift	1.9–2 nm force-switch
ρ [g/cm^3]	0.99802(1)<br>0.998[17]	0.99803(1)
η_b [mPa s]	0.698(9)	0.698(4)
γ_{lv} [mJ/m^2]	54.379(98)[12]	60.260(60)[12]

Table S1: Properties of SPC/E water with two different LJ cutoff schemes: a potential shift with a cutoff of 0.9 nm (as used in the original SPC/E publication, Ref. 17), and force switching between 1.9 and 2 nm (as used elsewhere in this work). Where no reference is given, quantities were calculated by us.

The water is driven along the x-direction, tangential to the SAM by a gravity-like force, where a force is applied to each liquid atom that is proportional to its mass. In the bulk, the liquid density is constant, which gives a constant force density and a quadratic flow profile with the vertex of the parabola at the liquid–vapor interface, i.e., a half-Poiseuille flow.

The surface–liquid force on each liquid atom is needed in order to extract the surface–liquid friction force as a function of liquid position, $f_f(z)$. This is extracted by carrying out two reruns with modified topologies. In both reruns, all bonded interactions are set to zero. In the first, all nonbonded interactions are left active, giving the total nonbonded force on each liquid atom. Here, we denote the total nonbonded force on the ith liquid atom as F_i. In the second, all nonbonded interactions involving the surface atoms are set to zero, which gives the nonbonded force on the ith liquid atom from just the other liquid atoms $F_i^{\text{liq. liq.}}$. Then the force on the ith liquid atom from the surface only is given by

$$F_i^{\text{surf. liq.}} = F_i - F_i^{\text{liq. liq.}} \,. \tag{S2}$$

Note that the *total* surface–liquid force can be obtained without reruns because

$$\sum_i F_i^{\text{liq. liq.}} = 0 \,. \tag{S3}$$

It follows from eq (S2) that to obtain $F_i^{\text{surf. liq.}}$, only a single rerun with the liquid–liquid interactions, $F_i^{\text{liq. liq.}}$, turned off would suffice. However, GROMACS does not allow for the pairwise definition of electrostatic interactions, instead allowing control only over atomic partial charges. Thus, deactivating liquid–liquid electrostatic interactions would require setting all partial charges in the liquid to zero, which would also deactivate the desired surface–liquid electrostatic interactions. This is why the two-rerun method is necessary.

S2 Position-Dependent Surface–Liquid Friction

Consider a system consisting of a fixed solid surface parallel to the xy-plane with an adsorbed liquid phase flowing over the surface along the x-direction in a steady-state flow. The friction between the surface and liquid is traditionally characterized by the Navier friction coefficient λ, defined via

$$F_f = -\lambda u_{\text{slip}} \,, \tag{S4}$$

where F_f is the tangential surface–liquid friction stress and u_{slip} the liquid slip velocity, i.e., the velocity of the liquid directly adjacent to the surface, relative to the surface.[19] Note that F_f and u_{slip} are both restricted to the x-direction here. At subnanometer scales, F_f acts on the liquid over a finite range of z-values,

$$F_f = \int \mathrm{d}z \, f_f(z) \,, \tag{S5}$$

where $f_f(z)$ is the friction force density in the x-direction at z due to just the surface (and *not* to adjacent liquid). In the linear-friction regime, a position-dependent steady-state friction coefficient $l(z)$ can be defined via

$$f_f(z) = -l(z)u(z) \,, \tag{S6}$$

where $u(z)$ is the velocity of the liquid in the x-direction at z. Here, $l(z)$ is itself taken to be local, i.e., friction at z is a function only of velocity at z and not at other nearby positions. This is validated by the accuracy of modeling of interfacial flow for many surface types where a local friction coefficient $l(z)$ is assumed, as shown in Figure S6. Combining eqs S4, S5 and S6 yields

$$\lambda u_{\text{slip}} = \int \mathrm{d}z \, l(z)u(z) \,. \tag{S7}$$

On the linear level, eq S7 holds for arbitrary profiles $u(z)$, including constant $u(z)$, with $u = u_{\text{slip}}$, from which follows an expression for the Navier friction coefficient in terms of the microscopically defined friction-coefficient profile,

$$\lambda = \int \mathrm{d}z \, l(z) \,. \tag{S8}$$

Together, eqs S7 and S8 give the slip velocity as a weighted mean of $u(z)$,

$$u_{\text{slip}} = \frac{\int \mathrm{d}z \, l(z)u(z)}{\int \mathrm{d}z \, l(z)} \,, \tag{S9}$$

for which eq S4 holds, even for position-dependent surface–liquid friction. For the limiting case of a sharply localized friction coefficient acting at one position z_{int} only, i.e., $l(z) \propto \delta(z - z_{\text{int}})$, eq S8 implies that $l(z) = \lambda\delta(z - z_{\text{int}})$, which together with eq S9 in turn implies that $u_{\text{slip}} = u(z_{\text{int}})$. Thus, we recover eq S4 under the classical assumptions of Navier, and eqs S8 and S9 can be thought of as giving generalizations of the Navier friction coefficient λ and slip velocity u_{slip}, respectively.

Combining eqs S6 and S8 yields a useful equation for the Navier friction coefficient in the case of a steady state flow,

$$\lambda = -\int \mathrm{d}z \, \frac{f_f(z)}{u(z)} \,. \tag{S10}$$

Equation S10 allows λ to be calculated directly from a nonequilibrium molecular dynamics (NEMD) simulation where a liquid is driven by a time-independent external force tangential to a fixed surface.

S3 Navier–Stokes and Stokes Equations for Position-Dependent Viscosity

The ith component of the momentum-balance equation for an arbitrary volume V reads

$$\frac{d}{dt}\int_V \mathrm{d}\boldsymbol{r}\, \rho u_i + \int_{\partial V} \mathrm{d}S_j \, \rho u_i u_j = \int_V \mathrm{d}\boldsymbol{r}\, f_i + \int_{\partial V} \mathrm{d}S_j \, \sigma_{ij} \,. \tag{S11}$$

The four terms, starting on the left, give the change in momentum, momentum flux, external force, and internal force on a surface element. The Einstein summation convention is used and arguments are omitted from the density $\rho(\boldsymbol{r}, t)$, velocity $\boldsymbol{u}(\boldsymbol{r}, t)$, force density $\boldsymbol{f}(\boldsymbol{r}, t)$, and stress tensor $\sigma_{ij}(\boldsymbol{r}, t)$ for brevity. Gauss's law may be used to write the surface integrals in S11 as volume integrals over divergences, yielding

$$\int_V \mathrm{d}\boldsymbol{r} \left(\frac{\partial}{\partial t}[\rho u_i] + \nabla_j [\rho u_i u_j] - f_i - \nabla_j \sigma_{ij} \right) = 0 \,. \tag{S12}$$

This holds for arbitrarily defined volumes V, so

$$\begin{aligned} f_i + \nabla_j \sigma_{ij} &= \frac{\partial}{\partial t}[\rho u_i] + \nabla_j [u_j \rho u_i] \\ &= \rho \dot{u}_i + u_i \dot{\rho} + u_i \nabla_j [u_j \rho] + u_j \rho \nabla_j u_i \,. \end{aligned} \tag{S13}$$

Using conservation of mass, $\dot{\rho} + \nabla_j u_j \rho = 0$, to cancel terms in S13, it follows that

$$f_i + \nabla_j \sigma_{ij} = \rho\left(\dot{u}_i + u_j \nabla_j u_i\right) \equiv \rho \frac{Du_i}{Dt} \,, \tag{S14}$$

where D/Dt is the material or substantial derivative,

$$\frac{D}{Dt} \equiv \frac{\partial}{\partial t} + u_j \nabla_j \,, \tag{S15}$$

which describes the change of a quantity for an observer comoving with the flow $\boldsymbol{u}$. The fluid stress tensor, σ_{ij}, can only depend on spatial derivatives of the velocity and not on the velocity directly. Further, assuming a Newtonian fluid, σ_{ij} must be rotationally invariant to linear order. We consider the case where the volume and shear viscosities are assumed to be homogeneous in time, but position-dependent, nonlocal (i.e., stress at $\boldsymbol{r}$ is also a function of motion at other positions $\boldsymbol{r}-\boldsymbol{r}'$), and having memory (i.e., stress at time t is also a function of motion at earlier times $t-t'$). In this case, the stress tensor σ_{ij} is given by the linear stress constitutive equation

$$\begin{aligned}\sigma_{ij}(\boldsymbol{r},t) &= -p(\boldsymbol{r},t)\delta_{ij} \\ &+ \int \mathrm{d}\boldsymbol{r}'\int_0^\infty \mathrm{d}t' \Bigg[\delta_{ij}\xi^\dagger(\boldsymbol{r},\boldsymbol{r}',t')\nabla_k u_k(\boldsymbol{r}-\boldsymbol{r}',t-t') \\ &+ \eta^\dagger(\boldsymbol{r},\boldsymbol{r}',t')\Big(\nabla_i u_j(\boldsymbol{r}-\boldsymbol{r}',t-t') \\ &+ \nabla_j u_i(\boldsymbol{r}-\boldsymbol{r}',t-t') \\ &- \frac{2}{3}\delta_{ij}\nabla_k u_k(\boldsymbol{r}-\boldsymbol{r}',t-t')\Big)\Bigg],\end{aligned} \tag{S16}$$

where $p(\boldsymbol{r},t)$ is the pressure and $\xi^\dagger(\boldsymbol{r},\boldsymbol{r}',t')$ and $\eta^\dagger(\boldsymbol{r},\boldsymbol{r}',t')$ are the volume- and shear-viscosity kernels, respectively.[20] The terms in eq S16 including p and $\xi^\dagger$ are isotropic (diagonal), giving the stress from static pressure and isotropic expansion, respectively. The term including $\eta^\dagger$ is deviatoric (traceless), giving the stress due to shear. A velocity gradient $\nabla_\alpha u_\beta(\boldsymbol{r}-\boldsymbol{r}',t-t')$ may be expanded around $(\boldsymbol{r}',t') = (\vec{0},0)$, giving

$$\begin{aligned}\nabla_\alpha u_\beta(\boldsymbol{r}-\boldsymbol{r}',t-t') =& \nabla_\alpha u_\beta(\boldsymbol{r},t) \\ &- r'_\gamma \nabla_\alpha\left[\nabla_\gamma u_\beta(\boldsymbol{r},t)\right] \\ &- t'\nabla_\alpha \dot{u}_\beta(\boldsymbol{r},t) \\ &+ \mathcal{O}\left((\boldsymbol{r}')^2,(t')^2,\boldsymbol{r}'t'\right).\end{aligned} \tag{S17}$$

Approximating $\nabla_\alpha u_\beta(\boldsymbol{r}-\boldsymbol{r}',t-t')$ by just the leading-order term, σ_{ij} may be written as

$$\begin{aligned}\sigma_{ij}(\boldsymbol{r},t) &= -p(\boldsymbol{r},t)\delta_{ij} + \xi(\boldsymbol{r})\delta_{ij}\nabla_k u_k(\boldsymbol{r},t) \\ &+ \eta(\boldsymbol{r})\left(\nabla_i u_j(\boldsymbol{r},t) + \nabla_j u_i(\boldsymbol{r},t) - \frac{2}{3}\delta_{ij}\nabla_k u_k(\boldsymbol{r},t)\right),\end{aligned} \tag{S18}$$

where $\xi(\boldsymbol{r})$ and $\eta(\boldsymbol{r})$ are position-dependent volume- and shear viscosities, given by

$$\begin{aligned}\xi(\boldsymbol{r}) &= \int \mathrm{d}\boldsymbol{r}'\int_0^\infty \mathrm{d}t'\,\xi^\dagger(\boldsymbol{r},\boldsymbol{r}',t') \quad \text{and} \\ \eta(\boldsymbol{r}) &= \int \mathrm{d}\boldsymbol{r}'\int_0^\infty \mathrm{d}t'\,\eta^\dagger(\boldsymbol{r},\boldsymbol{r}',t').\end{aligned} \tag{S19}$$

Note that although the viscosities ξ and η in eq (S19) are, in general, functions of the position vector $\boldsymbol{r}$, for the systems considered in this work, which are translationally invariant in x and y, they depend only on the coordinate z. The memory of the volume- and shear-viscosity kernels $\xi^\dagger$ and $\eta^\dagger$ (i.e., their t' dependence) can be fully accounted for in eq S18 via $\xi(\boldsymbol{r})$ and $\eta(\boldsymbol{r})$ because only steady-state systems are studied in this work, which causes terms in eq S17 that contain time derivatives (e.g. $t'\nabla_\alpha \dot{u}_\beta(\boldsymbol{r},t)$) to vanish.

The nonlocality of the viscosity kernels (i.e., their $\boldsymbol{r}'$ dependence) is similarly accounted for in eq S18 via $\xi(\boldsymbol{r})$ and $\eta(\boldsymbol{r})$ when higher-order terms in eq S17 that contain additional spatial derivatives (e.g. $r'_\gamma\nabla_\alpha\left[\nabla_\gamma u_\beta(\boldsymbol{r},t)\right]$) can be neglected. This is only valid in the context of eq S16 when the viscosity kernels decay rapidly for increasing $|\boldsymbol{r}'|$ on a length scale small compared to the spatial variation of the shear rate. For example, in the limiting case of a fluid where the shear is not only in a steady-state, but is also constant in space, eq S18 is exact. As the viscosity is mediated by fluid–fluid intermolecular interactions, the viscosity kernels can be expected to decay on length scales similar to the range of these interactions.[21] In this work, the rapid decay of the viscosity kernels is hypothesized *a priori*, and this hypothesis is well supported by the accuracy of modeling of interfacial flow for many surface types, as shown in Figure S6.

Calculating $\nabla_j\sigma_{ij}$ from eq S18 and substituting the result into eq S14 gives a formulation of the Navier–Stokes equation for position-dependent viscosity,[19,22,23]

$$\begin{aligned}\rho\frac{Du_i}{Dt} &= \rho\dot{u}_i + \rho u_j\nabla_j u_i \\ &= f_i - \nabla_i p + \nabla_i\left[\left(\xi(\boldsymbol{r}) - \frac{2\eta(\boldsymbol{r})}{3}\right)\nabla_j u_j\right] \\ &+ \nabla_j\left[\eta(\boldsymbol{r})\nabla_i u_j\right] + \nabla_j\left[\eta(\boldsymbol{r})\nabla_j u_i\right],\end{aligned} \tag{S20}$$

where arguments $\boldsymbol{r}$ and t are again omitted from ρ, u_i, f_i, and p for brevity. Consider the scaling relations $\rho u_j\nabla_j u_i \sim \rho u^2/L$ and $\eta\nabla_k\left[\nabla_k u_i\right] \sim \eta u/L^2$, where u and L are the characteristic speed and length, respectively. The nonlinear term, $\rho u_j\nabla_j u_i$, is negligible if

$$\frac{\rho u^2/L}{\eta u/L} = \frac{\rho u L}{\eta} \equiv \mathrm{Re} < 1\,, \tag{S21}$$

i.e., if the Reynolds number, Re, is small. For water, $\rho \approx 10^3$ kg/m^3 and $\eta \approx 10^{-3}$ kg/(m s), so Re < 1 if $uL < 10^{-6}$ m^2/s, which is fulfilled for the nanoscopic systems of interest to us. We note that for tangential flow on a planar surface, the term $u_j\nabla_j u_i$ vanishes even for high Reynolds numbers. Omission of the nonlinear term gives the Stokes' equation,

$$\begin{aligned}\rho\dot{u}_i &= f_i - \nabla_i p + \nabla_i\left[\left(\xi(\boldsymbol{r}) - \frac{2\eta(\boldsymbol{r})}{3}\right)\nabla_j u_j\right] \\ &+ \nabla_j\left[\eta(\boldsymbol{r})\nabla_i u_j\right] + \nabla_j\left[\eta(\boldsymbol{r})\nabla_j u_i\right].\end{aligned} \tag{S22}$$

S4 Calculation of Position-Dependent Viscosity

Consider a liquid phase, the behavior of which is described by eq S22, which is the Stokes equation for position-dependent shear and bulk viscosities $\eta(\boldsymbol{r})$ and $\xi(\boldsymbol{r})$. Here, ρ denotes the liquid mass density, u the liquid velocity, $\dot{u}$ its time derivative, and f the external force density exerted on the liquid, and the Einstein summation convention is used. Assume the system is translationally invariant in the xy-plane. It immediately follows that $\eta(\boldsymbol{r})$ depends only on z, so we denote it simply as $\eta(z)$. Next it is assumed that the flow is laminar and parallel, and the applied force $f = f_x(z,t)$ is constant over x and y and acts only in the x direction. It follows that $u = u_x(z,t)$ and $p = p(z,t)$. These symmetries cause several terms in eq S22 to vanish, leaving

$$\rho \dot{u}_x(z,t) = f_x(z,t) + \partial_z \left[\eta(z)\partial_z u_x(z,t)\right] . \tag{S23}$$

Next, we assume a steady state flow. Omitting the subscripts x for clarity gives

$$f(z) = -\partial_z \left[\eta(z)\partial_z u(z)\right] . \tag{S24}$$

Now consider a system consisting of a planar surface in the xy-plane with an adsorbed liquid slab. Letting z_0 denote a position below the liquid phase, integration of eq S24 from z_0 to an arbitrary z gives

$$\begin{aligned}\int_{z_0}^{z} \mathrm{d}z'\, f(z') &= -\int_{z_0}^{z} \mathrm{d}z'\, \partial_{z'} \left[\eta(z')\partial_{z'} u(z')\right] \\ &= -\eta(z)\partial_z u(z) .\end{aligned} \tag{S25}$$

Rearranging this equation gives an expression for the viscosity profile

$$\eta(z) = -\frac{1}{\partial_z u(z)} \int_{z_0}^{z} \mathrm{d}z'\, f(z') , \tag{S26}$$

which can be calculated numerically from driven-flow NEMD simulation data. Here, $f(z)$ consists of the sum of the applied driving force density $f_a(z)$ and surface-friction force density $f_f(z)$, and $\partial_z u(z)$ is the local liquid shear rate, which can be obtained numerically from the velocity profile using finite differences. In the steady state, the cumulative external stresses on liquid below z must be balanced by the liquid–liquid stress at z, i.e.

$$\sigma_{xz}^{\mathrm{ll}}(z) + \int_{z_0}^{z} \mathrm{d}z'\, f(z') = 0 . \tag{S27}$$

Together with eq (S26), this gives the more familiar Newton's law of viscosity (eq 3 from the main text),

$$\eta(z) = \frac{\sigma_{xz}^{\mathrm{ll}}(z)}{\partial_z u(z)} . \tag{S28}$$

S5 Fits of Velocity Profile Tails

In the driven-flow SAM/water simulations, both the average velocity and density of the water tend to decay toward zero as the SAM surface is approached. A lower liquid density means less data for velocity calculations, which results in noisy velocity profiles. Our remedy is to fit the decaying velocity profile tails with exponential functions. Such fits are shown in Figure S2. The upper block of plots are from simulations with a faster driven flow, where the applied driving stress F_a is greater, and are used for extracting viscosity profiles. The lower block are from simulations with a slower driven flow, i.e., with a smaller applied driving stress F_a, and are used for extracting surface–liquid friction coefficients. Each fit is carried out in just a 1-Å window, which is indicated in the respective plot by a shaded blue area. The velocity profile to the left of the center of the window (i.e., left of the vertical red line) is replaced by the fit values for the purpose of calculating the viscosity or friction-coefficient profiles. Also shown in each panel are the mass-density profiles for the liquid and surface, scaled by an arbitrary factor, as a positional reference.

S6 Calculation of Friction-Coefficient and Viscosity Profiles

Figure S3 shows, for all systems, the raw velocity $u(z)$ and surface–liquid friction force $f_f(z)$ data from driven-flow NEMD simulations used to extract the surface–liquid friction-coefficient profiles $l(z)$, which in turn are shown in the lower panels of the subfigures, directly below the corresponding raw data. Here, after the fitting procedure illustrated in Figure S2, $u(z)$ and $f_f(z)$ are both smoothed by convolution with the same Gaussian kernel with standard deviation σ_{sm}^{l}. For $l(z)$, the flow speed must be slower than for $\eta(z)$ to remain in the linear regime, especially for the more hydrophobic surfaces (see Section S8). For each system, the driving stress F_a^{η} applied for extracting $\eta(z)$ is chosen to be four times greater than F_a^{l}, the applied driving stress for extracting $l(z)$ for that system. Therefore, longer trajectories are required to obtain comparable $f_f(z)$ profiles, and $f_f(z)$ benefits more from noise reduction here than in the case of the $\eta(z)$ calculation. Profiles $l(z)$ are shown for various degrees of smoothing, as parametrized by σ_{sm}^{l}. The $l(z)$ data for $\sigma_{\mathrm{sm}}^{l} = 0.1$ Å, shown here as a red curve, are those reproduced in the main text and used in subsequent modeling and analysis.

Similarly, Figure S4 shows, for all systems, $u(z)$ and $f_f(z)$ data from similar, but faster, driven-flow simulations, used to extract the viscosity profiles $\eta(z)$, which are also shown in the lower panels of the subfigures,

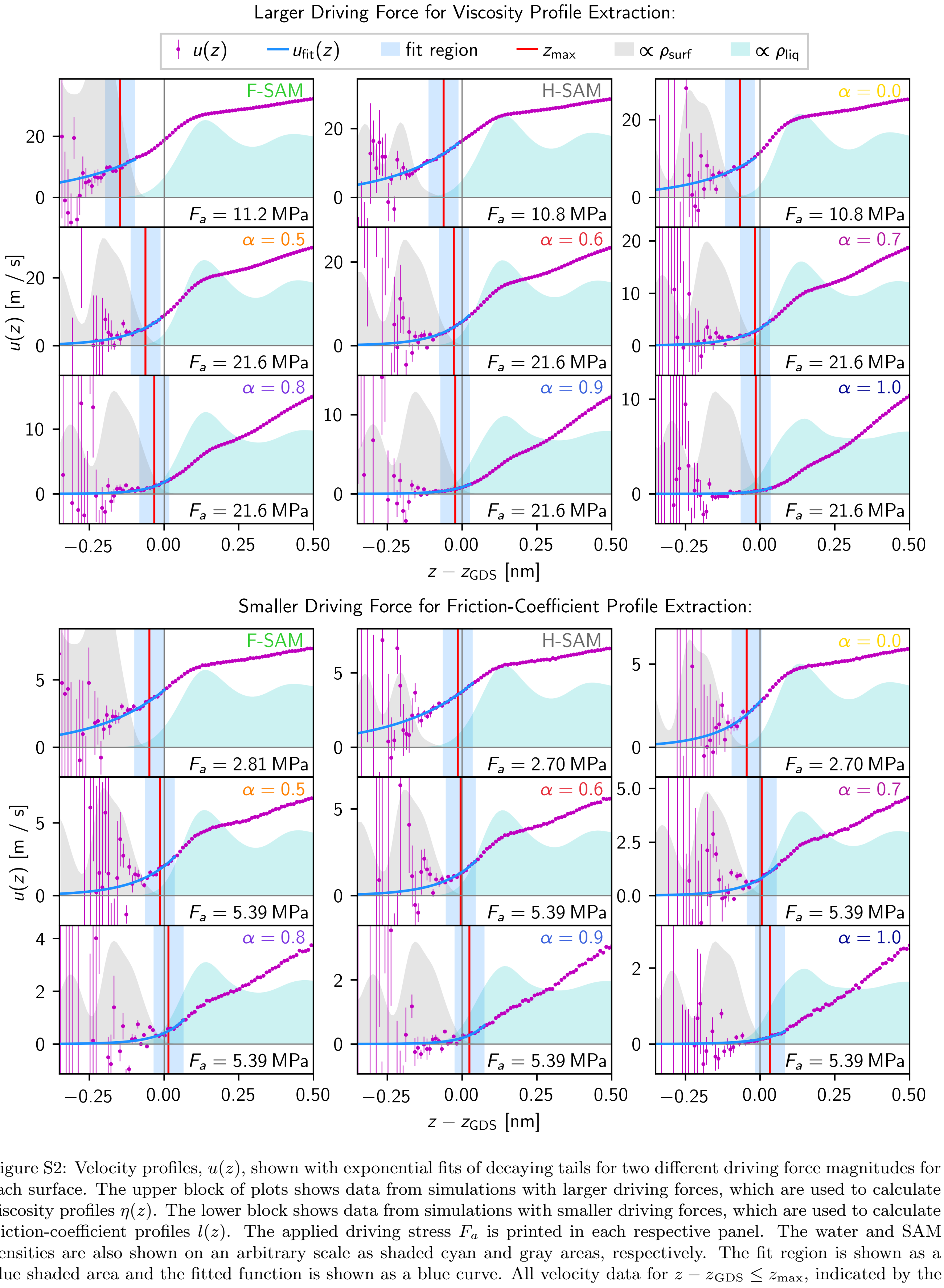

Figure S2: Velocity profiles, $u(z)$, shown with exponential fits of decaying tails for two different driving force magnitudes for each surface. The upper block of plots shows data from simulations with larger driving forces, which are used to calculate viscosity profiles $\eta(z)$. The lower block shows data from simulations with smaller driving forces, which are used to calculate friction-coefficient profiles $l(z)$. The applied driving stress F_a is printed in each respective panel. The water and SAM densities are also shown on an arbitrary scale as shaded cyan and gray areas, respectively. The fit region is shown as a blue shaded area and the fitted function is shown as a blue curve. All velocity data for $z - z_{\mathrm{GDS}} \leq z_{\max}$, indicated by the vertical red line, is replaced with the fit data for the calculation of viscosity or surface–liquid friction-coefficient profiles.

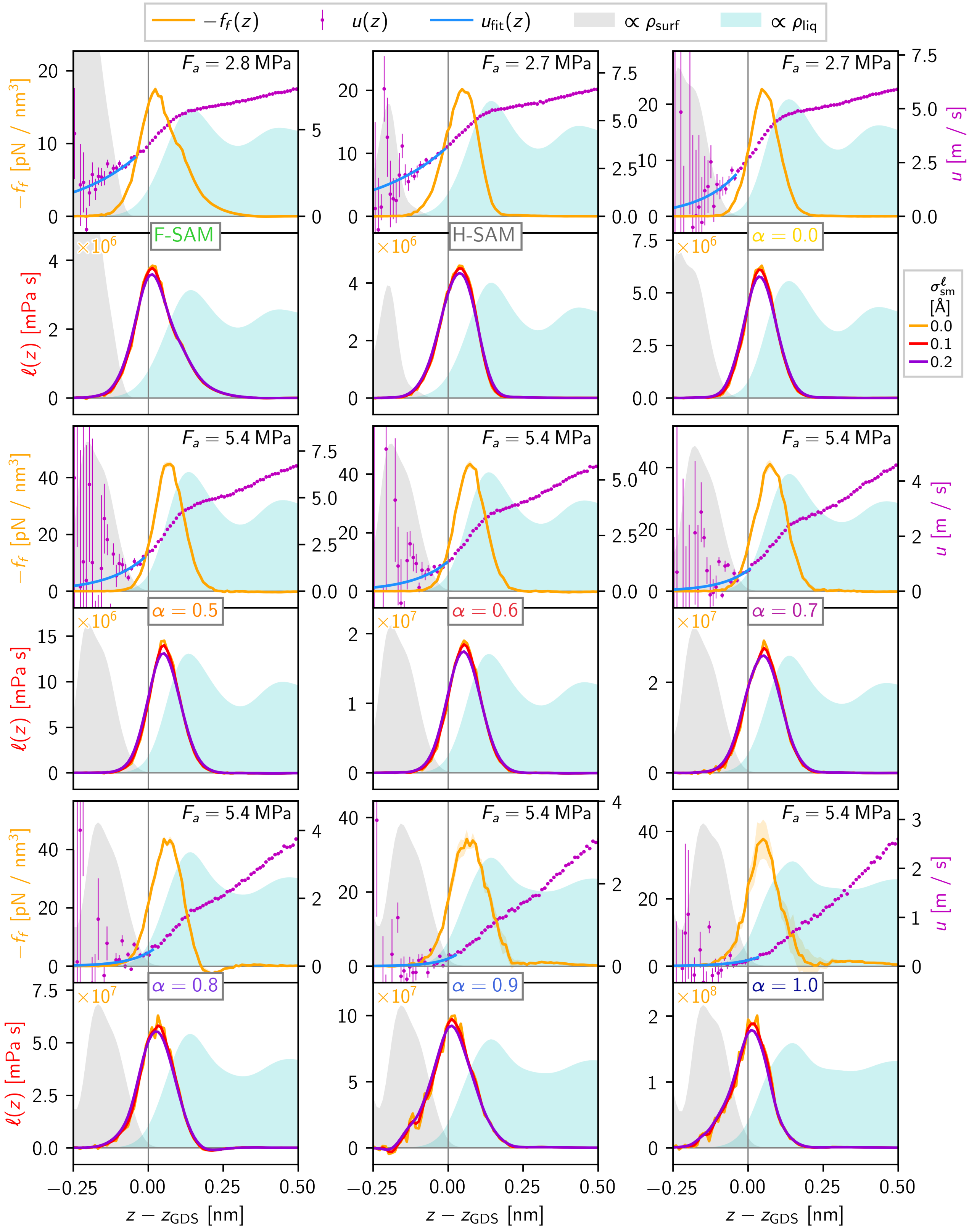

Figure S3: Raw velocity $u(z)$ and surface–liquid friction force $f_f(z)$ data (upper panels of each subfigure), and resulting friction-coefficient profiles $l(z)$ (lower) for all systems. Alongside the velocity profiles $u(z)$, the exponential functions fitted to the tails are shown. The friction-coefficient profiles $l(z)$ are shown for various degrees of smoothing of the input profiles $u(z)$ and $f_f(z)$. The water and SAM densities are also shown on an arbitrary scale as shaded cyan and gray areas, respectively.

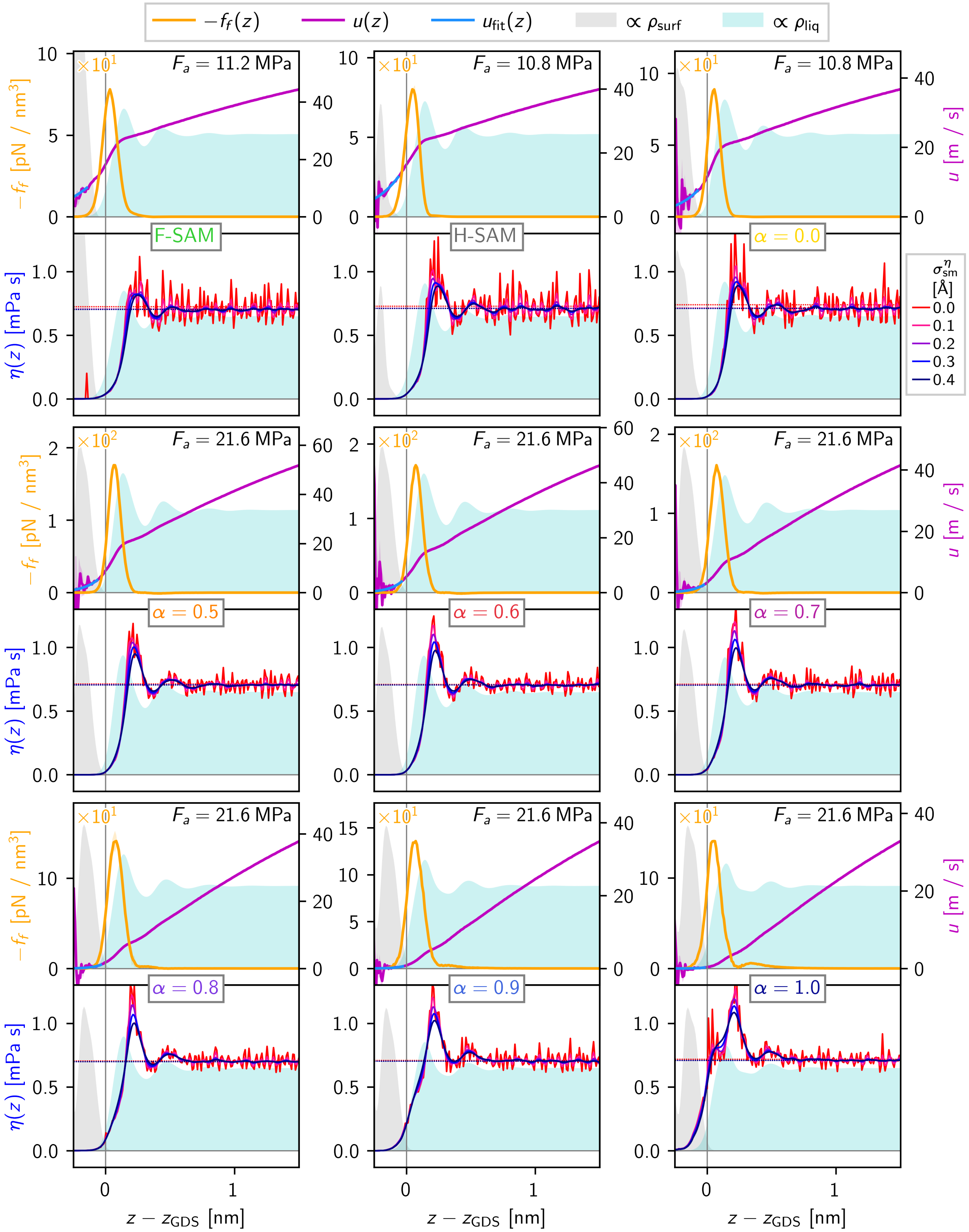

Figure S4: Raw velocity $u(z)$ and surface–liquid friction force $f_f(z)$ data (upper panels of each subfigure), and resulting viscosity profiles $\eta(z)$ (lower) for all systems. Alongside the velocity profiles $u(z)$, the exponential functions fitted to the tails are shown. The viscosity profiles are shown for various degrees of smoothing of just the input velocity profile $u(z)$. The water and SAM densities are also shown on an arbitrary scale as shaded cyan and gray areas, respectively.

directly below the corresponding raw data. Here, after the fitting procedure illustrated in Figure S2, just $u(z)$ is smoothed by convolution with a Gaussian kernel with standard deviation $\sigma^{\eta}_{\text{sm}}$. No data processing is applied to the input friction force profile $f_f(z)$ for calculating $\eta(z)$.

Smoothing of $u(z)$ is more important for calculation of $\eta(z)$, because $\eta(z)$ depends on the shear $\partial_z u(z)$, which is sensitively dependent on noise in the $u(z)$ data. Profiles $\eta(z)$ are shown for various degrees of smoothing, as parametrized by $\sigma^{\eta}_{\text{sm}}$. The data for $\sigma^{\eta}_{\text{sm}} = 0.3$ Å, shown here as a blue curve, are those reproduced in the main text and used in subsequent modeling and analysis. Here, it is apparent how the sharpness and height of the peaks in $\eta(z)$, especially in the first hydration layer, are attenuated by the smoothing.

In each panel of Figures S3 and S4, the corresponding mass-density profiles for the liquid and surface, scaled by an arbitrary factor, are shown as a positional reference. Also, the exponential fitting functions from Figure S2 are shown over just the domain where they replace the $u(z)$ data before smoothing.

S7 Numerically Solving the Stokes Equation

Consider a liquid layer bounded below by a flat solid surface and exposed above to a vapor phase, with both the solid–liquid and liquid–vapor interfaces having surface normal $\hat{z}$. Let a volume element of the liquid be acted upon by two force densities: a solid–liquid friction force density $f_f(z) = -l(z)u(z)$ and an applied driving force density $f_a(z)$. In a steady state, the flow is governed by eq 14 from the main text,

$$l(z)u(z) = f_a(z) + \partial_z \left[\eta(z)u'(z)\right] ,$$

where $u(z)$ is the velocity, $l(z)$ is the solid–liquid friction coefficient, $\eta(z)$ is the liquid shear viscosity, and $u'(z)$ indicates the derivative of $u(z)$ with respect to z. The product rule gives,

$$f_a(z) = l(z)u(z) - \eta'(z)u'(z) - \eta(z)u''(z) . \tag{S29}$$

Let the space along z be discretized into N points with interval h between them. We wish to solve for $u(z)$ in the discretized space, so we approximate the derivatives of $u(z)$ using central difference quotients,

$$f_a(z) \approx l(z)u(z) - \eta'(z)\frac{u(z+h) - u(z-h)}{2h} - \eta(z)\frac{u(z+h) - 2u(z) + u(z-h)}{h^2} . \tag{S30}$$

Indexing the discretization points by i and letting $f_i \equiv f(z_i)$ allows this to be written more compactly as

$$f^a_i \approx l_i u_i - \eta'_i \frac{u_{i+1} - u_{i-1}}{2h} - \eta_i \frac{u_{i+1} - 2u_i + u_{i-1}}{h^2} , \tag{S31}$$

where $f^a_i \equiv f_a(z_i)$. Grouping terms of u_i, u_{i+1} and u_{i-1} gives

$$f^a_i \approx \left(l_i + \frac{2\eta_i}{h^2}\right) u_i + \left(\frac{\eta'_i}{2h} - \frac{\eta_i}{h^2}\right) u_{i+1} + \left(-\frac{\eta'_i}{2h} - \frac{\eta_i}{h^2}\right) u_{i-1} , \tag{S32}$$

which is a set of N coupled linear equations. Here, η' is calculated numerically from η using a central difference scheme. Thus, only u is unknown. This can be written in terms of the matrix G as

$$\boldsymbol{f}^a \approx G\boldsymbol{u} . \tag{S33}$$

See that eq S33 can in principle be solved numerically for $\boldsymbol{u}$ in one step using a linear solver. Taking $i \in \{1, \ldots, N\}$, u_1 and u_N are also functions of u_0 and u_{N+1}, i.e., two boundary conditions (BCs) are also needed. Let the left (here, solid–liquid) and right (here, liquid–vapor) BCs be given by matrices G^L and G^R such that $G = G^0 + G^L + G^R$, with

$$\begin{aligned} G^0_{ii} &= l_i + \frac{2\eta_i}{h^2} , \\ G^0_{i(i+1)} &= \frac{\eta'_i}{2h} - \frac{\eta_i}{h^2} , \\ G^0_{i(i-1)} &= -\frac{\eta'_i}{2h} - \frac{\eta_i}{h^2} , \\ G^0_{ij} &= 0 \quad \text{for} \quad j \notin [i, i+1, i-1] . \end{aligned} \tag{S34}$$

As defined, $G^0\boldsymbol{u}$ contains all terms of the R.H.S. of eq S32 except the boundary terms proportional to u_0 and u_{N+1}. Thus, taking $G^L = \boldsymbol{0}$ is equivalent to assuming that $u_0 = 0$, i.e., it amounts to a zero-velocity BC. In addition to the zero-velocity BC, another obvious choice is a no-shear BC, where the derivative of the velocity vanishes. For the (left) solid–liquid boundary at u_1, letting $u'_1 = 0$ and approximating u'_1 with the backward-difference formula gives the condition $u_0 = u_1$, and

$$G^L_{ij} = \delta_{i1}\delta_{1j}\left(-\frac{\eta'_1}{2h} - \frac{\eta_1}{h^2}\right) , \tag{S35}$$

where δ_{ij} is the Kronecker delta. For the (right) liquid–vapor boundary, the no-shear BC can be obtained by a similar procedure, except that the forward-difference formula is used, and the added term is that for u_{N+1}, i.e.,

$$G^R_{ij} = \delta_{iN}\delta_{Nj}\left(\frac{\eta'_N}{2h} - \frac{\eta_N}{h^2}\right) . \tag{S36}$$

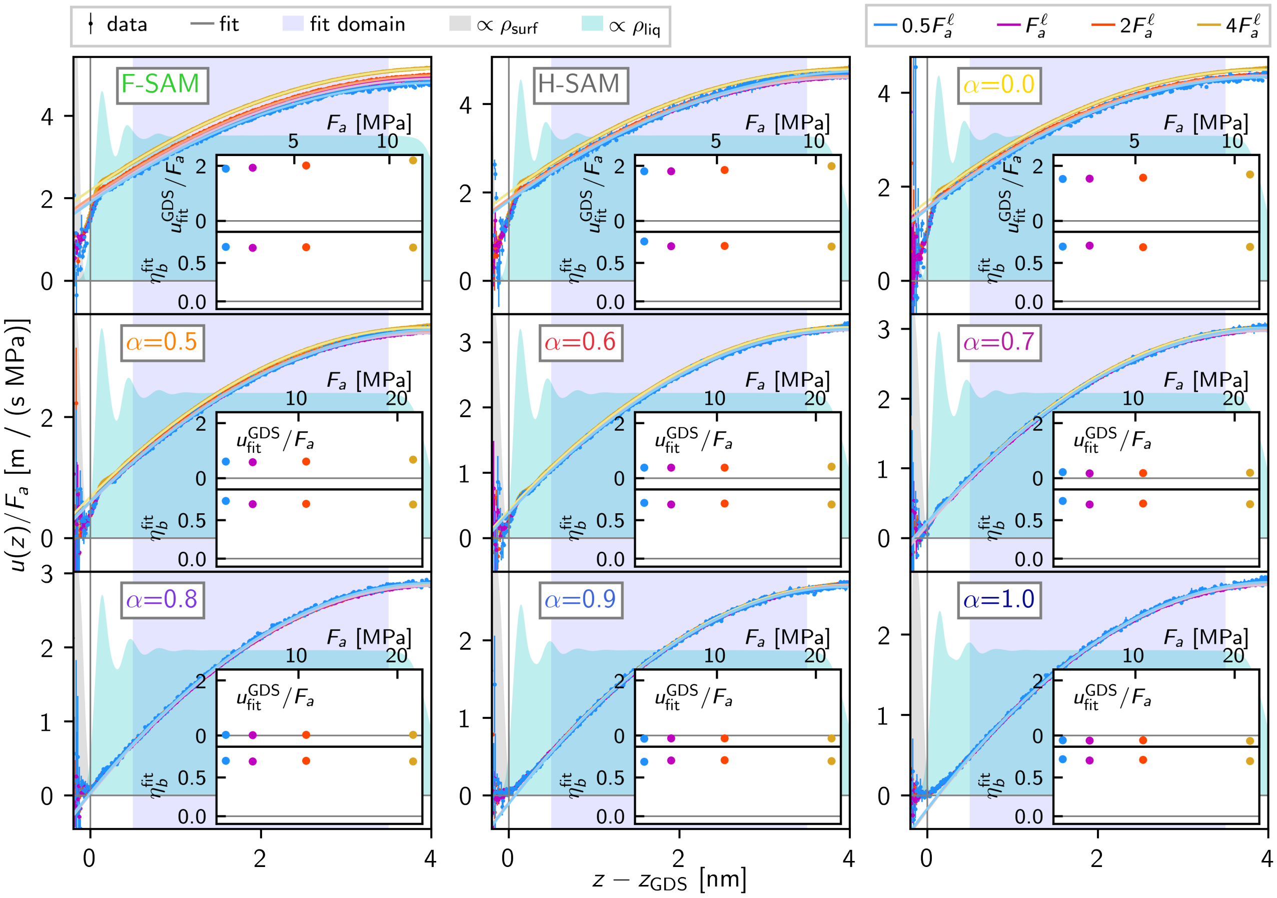

Figure S5: Velocity divided by applied driving stress, $u(z)/F_a$, for different driving stresses, shown alongside quadratic fits $u_{\mathrm{fit}}(z)/F_a$, where $u_{\mathrm{fit}}(z)$ is obtained by fitting eq S37 to $u(z)$ in the bulk domain, which is indicated by the blue shaded area. The water and SAM densities are also shown on an arbitrary scale as shaded cyan and gray areas, respectively. The upper insets plot $u_{\mathrm{fit}}(z_{\mathrm{GDS}})/F_a$, in units of m / (s MPa), over F_a. The lower insets show the bulk viscosity, in units of mPa s, calculated from the curvature of the fits via eq S38, over F_a.

In the case of the solid–liquid–vapor systems studied in this work, it is reasonable to take a zero-velocity BC on the left, near the solid–liquid interface ($G^L = \mathbf{0}$), and a no-shear BC on the right, near the liquid–vapor interface (G^R given by eq S36). These are the boundary conditions assumed wherever eq 14 is used to model velocity profiles, except in Section S10, where both zero-velocity *and* no-shear boundary conditions are explored at the solid–liquid interface.

S8 Verifying Linear-Response Regime by Velocity Profile Fits

To check if a system is in the linear-response regime for both the surface friction and viscosity, the quadratic function

$$u_{\mathrm{fit}}(z) = A\,(z - z_{\mathrm{GDS}})^2 + B\,(z - z_{\mathrm{GDS}}) + C\,, \quad \text{(S37)}$$

is fitted to the bulk-domain velocity profiles $u(z)$ of driven-flow NEMD simulations at four different driving stresses: $0.5F_a^l$, F_a^l, $2F_a^l$, and $4F_a^l = F_a^\eta$, where F_a^l is the driving stress applied to extract $l(z)$, and F_a^η the driving stress applied to extract $\eta(z)$, for that system. From the fit of eq S37, two quantities can be immediately calculated: the first is the shear viscosity in the bulk, given by

$$\eta_b^{\mathrm{fit}} = -\frac{f_a(z_b)}{2A}\,, \quad \text{(S38)}$$

where $f_a(z_b)$ is the driving force density in the fitted domain, and the second is the effective slip velocity at z_{GDS}, given by C.

Figure S5 plots the velocity profile $u(z)$ divided by the total applied driving stress F_a for each system studied in this work, at each of the four applied driving stresses. In the linear-response regime of both viscosity and friction, $u(z)/F_a$ should collapse onto a master curve for all driving stresses F_a. Outside the linear-response regime of the viscosity, the curvature of $u(z)/F_a$ should disagree for different values of F_a, while outside the linear-response regime of the surface–liquid

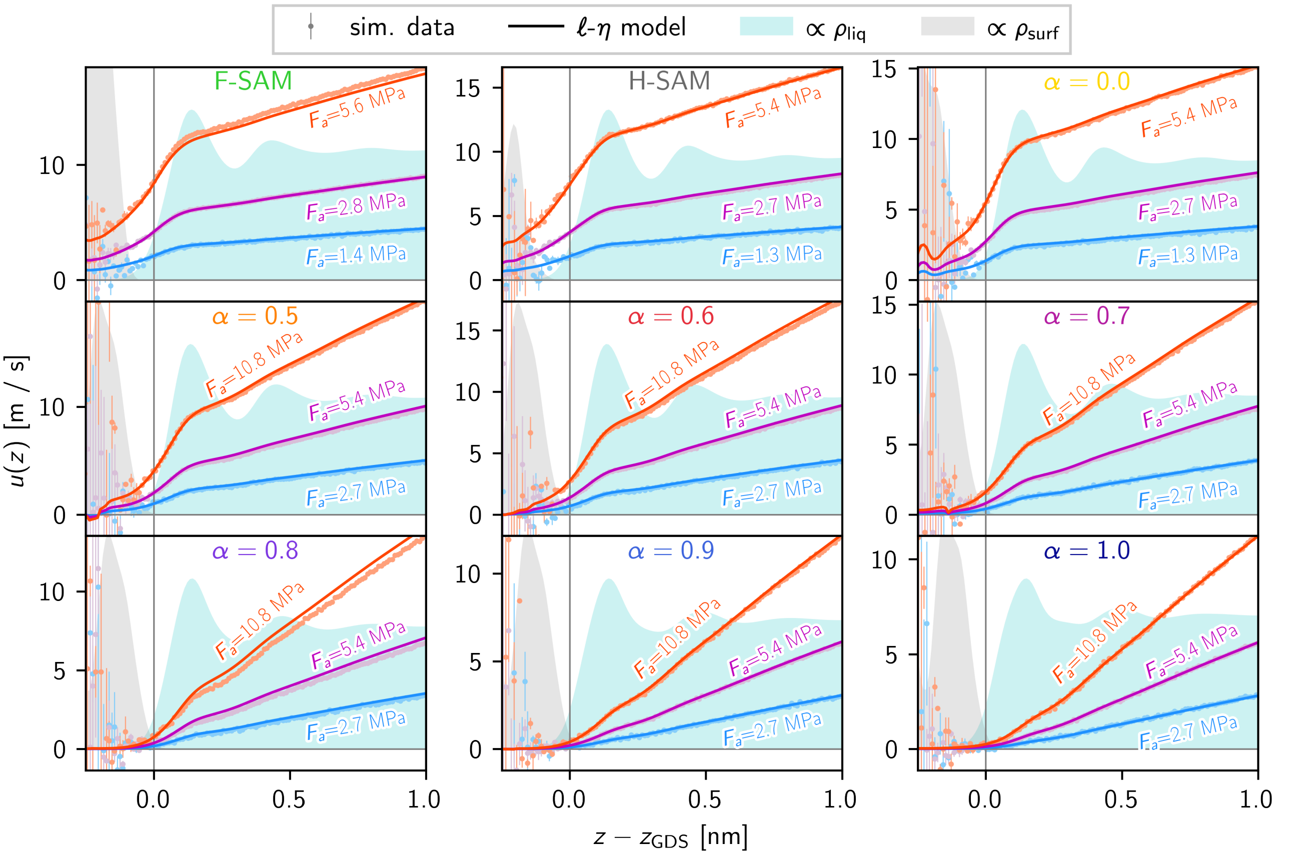

Figure S6: Velocity profiles $u(z)$ extracted from driven-flow simulations with different applied driving stresses F_a, compared with those calculated using the l-η model, i.e., by solving eq 14 from the main text numerically.

friction, $u(z)/F_a$ should disagree by a constant y-offset for different values of F_a. Also plotted in Figure S5 are $u_{\text{fit}}(z)/F_a$, where $u_{\text{fit}}(z)$ are the corresponding fits of eq S37. In each panel of Figure S5, the upper inset shows $u_{\text{fit}}(z_{\text{GDS}})/F_a$ plotted over F_a, and the lower inset shows η_b^{fit} (calculated via eq S38), also over F_a.

The data for the most hydrophilic systems agree very well across different values of F_a indicating that the systems are in the linear regime for both friction and viscosity. Moving to the more hydrophobic systems, $u(z)/F_a$ for the largest driving stress, $F_a = 4F_a^l$, is increasingly shifted along the y-axis, above the other lines. For the F-SAM/water system, the data for both the $F_a = 2F_a^l$ and $4F_a^l$ are shifted, indicating that the slip is in the linear regime only when $F_a \leq F_a^l$. We conclude that the friction-coeffient profiles $l(z)$ are all extracted in the linear regime of surface–liquid friction.

The viscosity data on the other hand, agree well across F_a, with the exception, for some systems, of the data for the smallest driving stresses, $F_a = 0.5F_a^l$, which appear to disagree mostly due to noise in the velocity. We conclude that the viscosity profiles $\eta(z)$ are also all extracted in the linear regime of the bulk viscosity.

S9 Modeling Flow for All Systems

For each system, driven-flow NEMD simulations are carried out at three applied driving stresses F_a. These are the driving stress F_a^l, used for the extraction of $l(z)$ for that respective system (as shown in Figure S3), as well as $0.5 \times F_a^l$ and $2 \times F_a^l$. In Figure S6, velocity profiles $u(z)$ from each of these simulations are compared with profiles calculated using the l–η model, i.e., by numerically solving eq 14 from the main text, using the $l(z)$ and $\eta(z)$ profiles plotted in Figure 2 in the main text, and taking the applied stress to be

$$f_a(z) = F_a \frac{\rho_{\text{liq}}(z)}{\int \mathrm{d}z' \, \rho_{\text{liq}}(z')} \,. \tag{S39}$$

The modeled velocity profiles agree remarkably well with the simulation data, which validates our approach, notably the locality of $l(z)$ and the neglect of higher-order gradient terms leading to the constitutive relation for $\eta(z)$, as described in Sections S2 and S3 and the main text, and indicates that $l(z)$ and $\eta(z)$ were extracted in the linear regime.

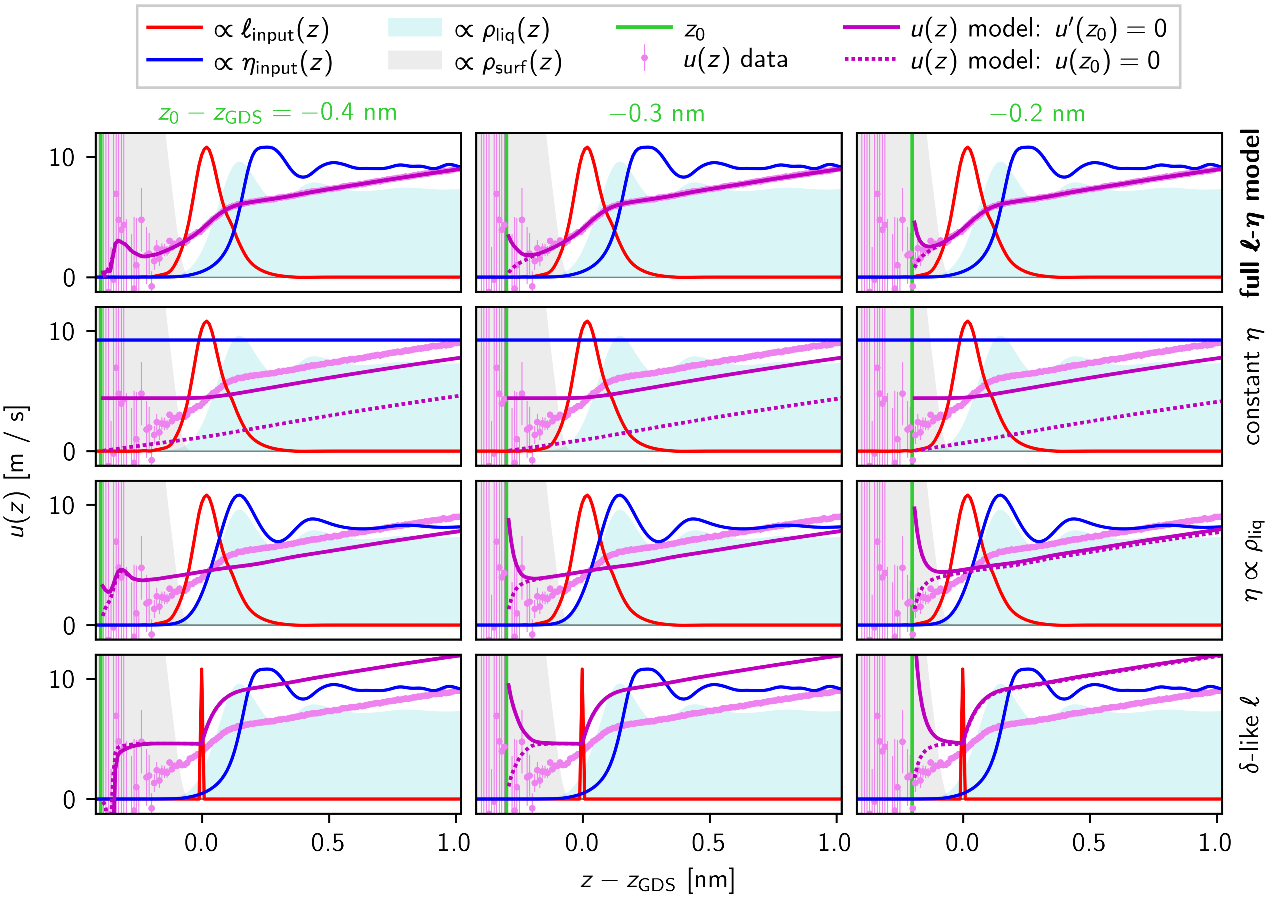

Figure S7: Comparison of different approaches to modeling the velocity profile of water on an F-SAM to a velocity profile extracted directly from a driven-flow NEMD simulation with a driving stress of 2.8 MPa (plotted as dots with error bars). Each row shows data for a different model (see the labels to the right of each row); the models are parametrized by the input surface–liquid friction-coefficient profiles $l_{\text{input}}(z)$ and viscosity profiles $\eta_{\text{input}}(z)$, which are also plotted with an arbitrary scaling factor. Each column shows data for a different lower domain boundary z_0 (see the labels at the top of each column). In each panel, the velocity profile is modeled with two different boundary conditions, $u'(z_0) = 0$ (solid line) and $u(z_0) = 0$ (dotted line). The mass-density profiles of the F-SAM and water are also shown as shaded areas as a positional reference with an arbitrary scaling factor.

S10 Other Approaches to Modeling Flow

We show in Figure S6 that the l–η model, i.e., solving eq 14 from the main text numerically using the extracted $l(z)$ and $\eta(z)$ profiles, is accurate for modeling nanoscopic flow near surfaces. This leaves open the question as to whether simpler approaches would predict the behavior equally as well. This is explored in Figure S7, where each panel plots the velocity profile $u(z)$ extracted from an F-SAM/water driven-flow simulation with driving stress $F_a = 2.8$ MPa, compared to a flow generated by a numerical model. Each row shows results from a different model, i.e, a solution of eq 14 from the main text with different simplifying assumptions about the input profiles $l_{\text{input}}(z)$ and $\eta_{\text{input}}(z)$. A finite domain along z must be chosen in which to solve the equation. We call the lower bound of this domain z_0, and the columns of Figure S7 correspond to three choices of z_0, all of which are below where $l(z)$ and $\eta(z)$ have decayed to near zero. We plot the data for different values of z_0 to check whether the modeled flow is dependent on z_0 or not. In each panel, there are two modeled velocity profiles plotted, a solid and a dotted line, which correspond to two different boundary conditions: $u'(z_0) = 0$ (no shear) and $u(z_0) = 0$ (zero velocity), respectively. At the upper boundary at $z - z_{\text{GDS}} = z_1 \approx 4$ nm, the no-shear boundary condition, $u'(z_1) = 0$, is always used.

The top row shows our full l–η model, which captures the velocity profile very accurately, for all values of z_0 and for both boundary conditions. There is slight disagreement near z_0, but this is in a zone with effectively zero liquid density and poor statistics.

The second row shows a simplified model where the

shear viscosity is assumed to be constant everywhere. The value taken is the shear viscosity from the bulk, η_b. This model fails to capture both the detailed interfacial behavior and the net slip of the liquid, reflected as a constant shift in velocity along the y-direction. For the $u(z_0) = 0$ boundary condition, the slip is also dependent on the choice of z_0.

The third row shows a simplified model where the viscosity is assumed to be proportional to the density of water molecules, scaled such that it approaches the correct viscosity in the bulk, η_b. This model fails very similarly to the constant viscosity model in the row above, though it seems to be independent of z_0.

The bottom row shows a model where only the Navier friction coefficient λ, rather than the entire $l(z)$ profile, is used. Here, the arbitrary assumption that the friction acts at the Gibbs dividing surface of the liquid is made. Like the complete l–η model, this model seems to be independent of z_0, but it again fails to capture both the detailed behavior near the interface and more importantly, the slip behavior.

Therefore, when modeling flow with the Stokes equation, accounting for the position-dependence of both the surface-friction and the viscosity, as the complete l–η model does, gives far more accurate results at subnanometer scales.

S11 Contact Angles

We measure the contact angles of all of our systems by simulating cylindrical droplets, which are continuous over the periodic boundary along the y-direction. Cylindrical droplets have been shown to be less susceptible to finite-size effects than spherical ones, and have the same contact angle in the macroscopic limit.[24] Figure S8 shows a snaphot of a 2048-molecule cylindrical droplet on an F-SAM. For each surface type, we simulate droplets of 2048, 4096, 8192, and 16384 water molecules and extract contact angles θ_μ from these. These "microscopic" contact angles θ_μ can deviate from the macroscopic contact angle θ due to finite-size effects. Thus, assuming a first-order correction to the work of adhesion,

$$\cos\theta_\mu = \cos\theta - \frac{C_0}{a}\,, \tag{S40}$$

where a is the footprint radius of the droplet, we extrapolate to the $1/a \to 0$ limit. In the case of the cylindrical droplets, a is half the footprint length along the x-direction. Extrapolations for all systems studied are shown in Figure S8. For the (α=1)-SAM, the water fully wetted the SAM, so no contact angle could be extracted.

The method we used for extracting contact angles was

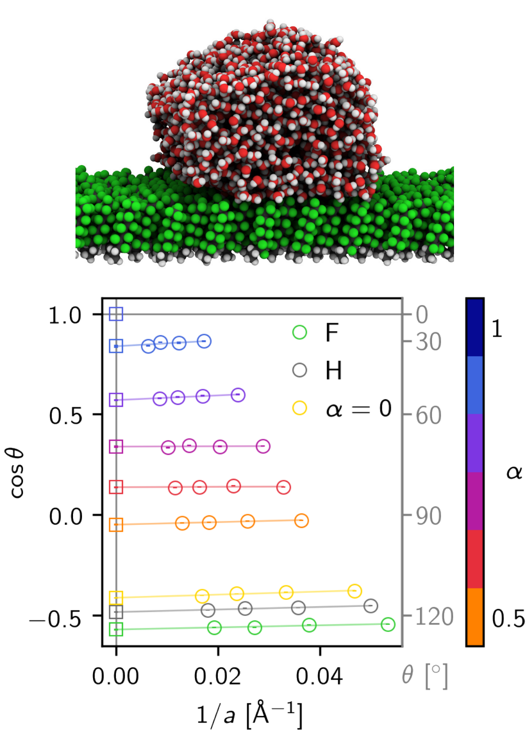

Figure S8: Extrapolation of microscopic contact angles to the macroscopic limit. Here, a is the droplet footprint radius. The circles indicate microscopic contact angles extracted from cylindrical droplet simulations, and the squares indicate the macroscopic value obtained by extrapolation of the linear fits, which are shown as solid lines, to $1/a = 0$. Shown above the plot is a snapshot of a cylindrical droplet of 2048 water molecules on an F-SAM (the smallest simulated droplet).

first published in Ref. 12 and we outline it briefly here. From each simulation, the mass-density profile $\rho_{\mathrm{drop}}(z)$ along the surface normal $\hat{z}$ of the liquid is extracted. The mass density of a planar liquid slab adsorbed on the same surface, $\rho_{\mathrm{slab}}(z)$, is also extracted from an equilibrium SAM/water-slab simulation. The droplet geometry can then be obtained by fitting the function

$$\frac{\rho_{\mathrm{drop}}(z)}{\rho_{\mathrm{slab}}(z)} = \frac{1}{2L_x}\times \int_0^{L_x} \mathrm{d}x \left(1 - \tanh\left(\frac{\sqrt{x^2 + (z - z_0)^2} - R_0}{d}\right)\right) \tag{S41}$$

to determine the parameters z_0, R_0, and d, which are the center-position of the droplet, droplet radius, and the sigmoid width d. Here, L_x is the system box length along the x-direction. The integral in eq S41 is evaluated numerically. The droplet radius R_0 is in fact the radius to the half-maximum position of the sigmoid, whereas the desired radius is the radius to the Gibbs dividing surface. These are the same for a flat interface, but not for a curved one. The Gibbs radius can

be calculated for a cylindrical droplet via

$$R_{\text{Gibbs}} = d\sqrt{\frac{-1}{2}\text{Li}_2\left(e^{-2R_0/d}\right)}\,, \tag{S42}$$

where Li_2 is the polylogarithm function. We approximate this by the Taylor expansion up to second order,

$$R_{\text{Gibbs}} \approx R_0\left(1+\frac{\pi^2}{24}\left(\frac{d}{R_0}\right)^2\right)\,. \tag{S43}$$

From the droplet center position z_0, the Gibbs radius R_{Gibbs}, and the position of the Gibbs dividing surface at the bottom of the planar liquid z_{Gibbs}, the contact angle can then be calculated from

$$\cos\theta = \frac{z_{\text{Gibbs}} - z_0}{R_{\text{Gibbs}}}\,. \tag{S44}$$

A more detailed derivation and discussion can be found in Ref. 12 and its supplement.

S12 Effective Viscosity Profiles

Consider a system with a surface–liquid interface parallel to the xy-plane, where the liquid is driven in the x-direction and has reached a steady-state flow, as described in Sections S2 and S4. Analogously to eq S27, the cumulative external stresses on the solid and liquid together below z must be balanced by the total intermolecular stress at z, i.e.,

$$\sigma^{\text{ll}}_{xz}(z) + \sigma^{\text{sl}}_{xz}(z) + \int_{z_0}^{z} \mathrm{d}z'\, f_a(z') = 0\,, \tag{S45}$$

where z_0 lies below the bottom of the combined solid–liquid system. Equation 4 from the main text reads

$$\eta_{\text{eff}}(z) = \frac{\sigma^{\text{ll}}_{xz}(z) + \sigma^{\text{sl}}_{xz}(z)}{\partial_z u(z)}\,, \tag{S46}$$

which, together with eq S45 yields

$$\eta_{\text{eff}}(z)\partial_z u(z) = -\int_{z_0}^{z} \mathrm{d}z'\, f_a(z')\,. \tag{S47}$$

The solid is subject to external constraint forces in the systems studied in this work, which contribute to $f_a(z)$ well below the solid-liquid interface. Thus, to simplify our calculations, eq S47 is rewritten as

$$\begin{aligned}\eta_{\text{eff}}(z)\partial_z u(z) &= -\int_{z_0}^{z_b} \mathrm{d}z'\, f_a(z') - \int_{z_b}^{z} \mathrm{d}z'\, f_a(z')\\ &= -\int_{z_0}^{z_b} \mathrm{d}z'\, f_a(z') + \int_{z}^{z_b} \mathrm{d}z'\, f_a(z')\,,\end{aligned} \tag{S48}$$

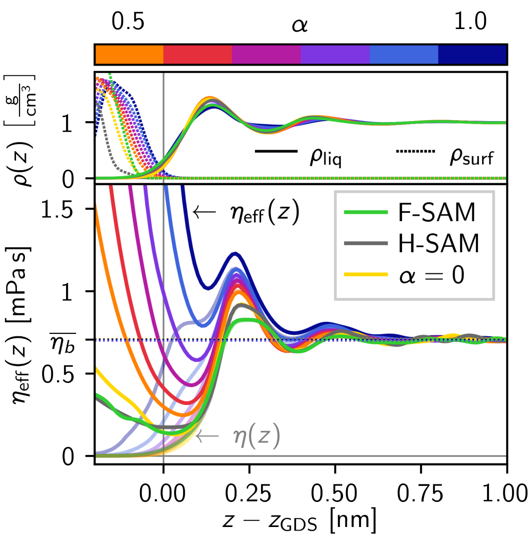

Figure S9: Effective viscosity profiles calculated via eq S50 for all systems studied in this work. Also plotted as translucent lines for comparison are the viscosity profiles calculated via eq S26.

where z_b is a position in the bulk of the liquid far from the solid surface. Comparison of eqs 3 and 4 from the main text, and the fact that the surface-liquid stress in the bulk vanishes, i.e., $\sigma^{\text{sl}}_{xz}(z_b) = 0$, reveals that $\eta_{\text{eff}}(z_b) = \eta(z_b) = \eta_b$. Together with eq S47, this yields

$$\eta_b\partial_{z_b} u(z_b) = -\int_{z_0}^{z_b} \mathrm{d}z'\, f_a(z')\,, \tag{S49}$$

which is in turn substituted into eq S48 to yield

$$\eta_{\text{eff}}(z) = \frac{1}{\partial_z u(z)}\left(\eta_b\partial_{z_b} u(z_b) + \int_{z}^{z_b} \mathrm{d}z'\, f_a(z')\right)\,. \tag{S50}$$

Equation S50 allows the calculation of $\eta_{\text{eff}}(z)$ without any knowledge of the surface–liquid interactions, making it much simpler to calculate than $\eta(z)$. For a steady-state Couette flow between oppositely moving parallel walls, where $f_a(z) = 0$ in the liquid, eq S50 reduces to

$$\eta_{\text{eff}}(z) = \frac{\eta_b\partial_{z_b} u(z_b)}{\partial_z u(z)} = -\frac{F_a}{\partial_z u(z)}\,, \tag{S51}$$

where F_a is the total stress from the driving force on the bottom wall. This formulation has been used in many previous works.[25,26]

Figure S9 shows effective viscosity profiles $\eta_{\text{eff}}(z)$, calculated via eq S50, for all systems studied in this work. Far from the surface, the effective viscosity profiles $\eta_{\text{eff}}(z)$ agree with the viscosity profiles $\eta(z)$, but diverge near the interface to account for the rapid decrease in flow there, which is in fact due to surface–liquid friction. This divergence in the effective viscosity profile has also be seen in previous works.[25–27]

S13 Bulk Shear Viscosity from the Green–Kubo Relation

In the linear-response regime, the Green–Kubo relation,

$$\widetilde{\eta}(\nu) = \beta V \int_0^\infty \mathrm{d}t\, e^{-i2\pi\nu t} \left\langle \sigma_{ij}(t)\sigma_{ij}(0)\right\rangle \,, \tag{S52}$$

gives the frequency-dependent viscosity $\widetilde{\eta}(\nu)$ from a simulation of bulk liquid with periodic boundary conditions, where $\sigma_{ij}(t)$ is any off-diagonal element of the stress tensor ($i \neq j$), $\beta = 1/(k_B T)$ is the inverse thermal energy, and V is the system volume.[28,29] Here, $\langle \ldots \rangle$ indicates a thermal average, and $\langle \sigma_{ij}(t)\sigma_{ij}(0)\rangle$ is the time autocorrelation function (ACF) of $\sigma_{ij}(t)$. However, *all* elements of σ_{ij} contain information about the shear viscosity, so eq S52 makes suboptimal use of the existing data for obtaining $\widetilde{\eta}(\nu)$. Let $\Pi_{ij}(t)$ be the deviatoric stress tensor,

$$\Pi_{ij}(t) = \sigma_{ij}(t) + \delta_{ij} p(t)\,, \tag{S53}$$

where p is the time-dependent pressure,

$$p(t) = -\frac{1}{3}\sum_{i=1}^{3} \sigma_{ii}(t)\,. \tag{S54}$$

See that $\Pi_{ij}(t)$ is traceless by construction, i.e.,

$$\Pi_{xx}(t) + \Pi_{yy}(t) + \Pi_{zz}(t) = 0\,. \tag{S55}$$

The volume viscosity, which parameterizes dissipation under compression, can be found via fluctuations of the pressure $p(t)$, while the shear viscosity, which parameterizes dissipation under shear, can be found via fluctuations of the nine elements of $\Pi_{ij}(t)$. Because $\Pi_{ij}(t)$ is symmetric, the number of independent elements is reduced by three. Equation S55 imposes a further constraint, reducing the total number of independent elements to five. These are conventionally the off-diagonal elements Π_{xy}, Π_{yz} and Π_{xz}, and two additional stresses constructed from the diagonal elements,[30]

$$Q_{xy} = \frac{\Pi_{xx} - \Pi_{yy}}{2} \quad \text{and} \qquad Q_{yz} = \frac{\Pi_{yy} - \Pi_{zz}}{2}\,. \tag{S56}$$

Using the tracelessness given by eq S55, it is easy to show that the diagonal elements can all be expressed in terms of Q_{xy} and Q_{yz},

$$\Pi_{xx} = \frac{4Q_{xy} + 2Q_{yz}}{3}\,, \qquad \Pi_{yy} = \frac{-2Q_{xy} + 2Q_{yz}}{3}\,, \qquad \Pi_{zz} = \frac{-2Q_{xy} - 4Q_{yz}}{3}\,,$$

i.e., Q_{xy} and Q_{yz} span the diagonal elements. The elements Q_{xy} and Q_{yz} are themselves off-diagonal elements of the stress tensor in frames of reference rotated by 45°. This can be expressed more concretely in terms of rotation matrices. The matrices for rotation about x and z are given by

$$R_x(\theta) = \begin{pmatrix} 1 & 0 & 0 \\ 0 & \cos\theta & \sin\theta \\ 0 & -\sin\theta & \cos\theta \end{pmatrix}\,, \qquad R_z(\theta) = \begin{pmatrix} \cos\theta & \sin\theta & 0 \\ -\sin\theta & \cos\theta & 0 \\ 0 & 0 & 1 \end{pmatrix}\,.$$

It is easy to show by matrix multiplication and using the symmetry of Π that

$$\left(R_z(-45°)\cdot \Pi \cdot R_z^T(-45°)\right)_{xy} = Q_{xy}\,,$$

and

$$\left(R_x(-45°)\cdot \Pi \cdot R_x^T(-45°)\right)_{yz} = Q_{yz}\,.$$

Thus, for an isotropic system, the ACFs of Q_{xy} and Q_{yz} are identical to those of the off-diagonal elements of Π_{ij}. Let $Q_{ij}^t \equiv Q_{ij}(t)$ and $\Pi_{ij}^t \equiv \Pi_{ij}(t)$. The ACF of Q_{ij}^t is given by

$$\left\langle Q_{ij}^t Q_{ij}^0 \right\rangle = \frac{1}{4}\left\langle (\Pi_{ii}^t - \Pi_{jj}^t)(\Pi_{ii}^0 - \Pi_{jj}^0)) \right\rangle = \frac{1}{4}\left(\left\langle \Pi_{ii}^t\Pi_{ii}^0\right\rangle + \left\langle \Pi_{jj}^t\Pi_{jj}^0\right\rangle - \left\langle \Pi_{ii}^t\Pi_{jj}^0\right\rangle - \left\langle \Pi_{jj}^t\Pi_{ii}^0\right\rangle\right)\,. \tag{S57}$$

Because of isotropy,

$$\left\langle \Pi_{ii}^t\Pi_{ii}^0\right\rangle = \left\langle \Pi_{jj}^t\Pi_{jj}^0\right\rangle \quad \text{and} \tag{S58}$$

$$\left\langle \Pi_{ii}^t\Pi_{jj}^0\right\rangle = \left\langle \Pi_{kk}^t\Pi_{ll}^0\right\rangle\,, \tag{S59}$$

for any i, j, k, and l. Thus, eq S57 reduces to

$$\left\langle Q_{ij}^t Q_{ij}^0 \right\rangle = \frac{1}{2}\left(\left\langle \Pi_{ii}^t\Pi_{ii}^0\right\rangle - \left\langle \Pi_{ii}^t\Pi_{jj}^0\right\rangle\right)\,. \tag{S60}$$

From the tracelessness of Π_{ii}, it follows that

$$\begin{aligned}\left\langle \Pi_{ii}^t\Pi_{jj}^0\right\rangle &= \left\langle \Pi_{ii}^t\left(-\Pi_{ii}^0 - \Pi_{kk}^t\right)\right\rangle \\ &= -\left\langle \Pi_{ii}^t\Pi_{ii}^0\right\rangle - \left\langle \Pi_{ii}^t\Pi_{kk}^0\right\rangle\,,\end{aligned} \tag{S61}$$

where $i \neq j$, $j \neq k$, and $i \neq k$. From isotropy,

$$\begin{aligned}\left\langle \Pi_{ii}^t\Pi_{jj}^0\right\rangle &= -\left\langle \Pi_{ii}^t\Pi_{ii}^0\right\rangle - \left\langle \Pi_{ii}^t\Pi_{jj}^0\right\rangle \\ \Rightarrow \quad \left\langle \Pi_{ii}^t\Pi_{jj}^0\right\rangle &= -\frac{1}{2}\left\langle \Pi_{ii}^t\Pi_{ii}^0\right\rangle\,.\end{aligned} \tag{S62}$$

Thus, eq S60 gives

$$4\left\langle Q_{ij}^t Q_{ij}^0\right\rangle = 3\left\langle \Pi_{ii}^t\Pi_{ii}^0\right\rangle\,. \tag{S63}$$

We wish to apply eq S52 to each of Π_{xy}, Π_{yz} and Π_{xz}, Q_{xy} and Q_{yz} and average the results. To minimize the effect of numerical errors in the stress tensor

elements from simulations, we average over ACFs of all off-diagonal elements, i.e., the average will include terms with ACFs of the elements Π_{xy}, Π_{yx}, Π_{yz}, Π_{zy}, Π_{xz}, and Π_{zx}. Because we effectively double count ACFs of Π_{ij} in the average, ACFs of Q_{ij} should also be double counted so that they are equally weighted, i.e., there should be a total of four ACFs of Q_{ij}. This is equivalent to exactly three ACFs of Π_{ii} according to eq S63. This is convenient, because all dimensions should be sampled equally, and there are three dimensions, i.e., ACFs of Π_{xx}, Π_{yy}, and Π_{zz} each contribute to the mean with a weight of 4/3. In all, there are ten equally contributing terms (four Q_{ij} ACFs and six Π_{ij} ACFs) in the sum, so a factor 1/10 is needed to obtain the mean. This gives, finally,

$$\widetilde{\eta}(\nu) = \frac{\beta V}{10} \int_0^\infty \mathrm{d}t \, e^{-i2\pi\nu t} \sum_{i,j} \langle \Pi_{ij}(t)\Pi_{ij}(0)\rangle \ . \quad \text{(S64)}$$

The factor 1/10 was first derived in Ref. 31.

The shear viscosity in the steady-state limit η_s may be found either by simply reading off $\widetilde{\eta}(\nu = 0)$, or equivalently, by taking the inverse Fourier transform of $\widetilde{\eta}(\nu)$ to obtain the viscosity $\eta(t)$ as a linear response function in the time domain,

$$\eta(t) = \frac{\beta V}{10} \sum_{i,j} \langle \Pi_{ij}(t)\Pi_{ij}(0)\rangle \ , \quad \text{(S65)}$$

and taking the time integral from $t = 0$ to ∞,

$$\eta_s = \frac{\beta V}{10} \int_0^\infty \mathrm{d}t \sum_{i,j} \langle \Pi_{ij}(t)\Pi_{ij}(0)\rangle \ . \quad \text{(S66)}$$

In practice, the simulated ACFs of Π_{ij} will be poorly behaved for long times due to insufficient data. Instead, the running integral can be plotted, and should clearly plateau after correlations have vanished if there is sufficient data overall. An average may then be taken over values in the plateau of the running integral.

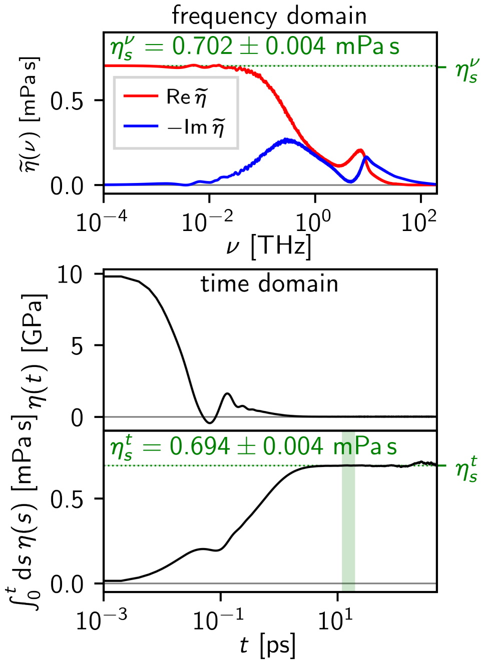

Figure S10: The viscosity spectrum calculated via eq S64 of an NpT simulation of 4096 SPC/E water molecules in bulk with periodic boundary conditions at 300 K and 1 bar. **Upper:** The real and imaginary parts of $\widetilde{\eta}(\nu)$ in the frequency domain. The zero-frequency value is shown as a dotted horizontal line and is denoted η_s^ν. **Middle:** The Fourier transform of $\widetilde{\eta}(\nu)$, which is the response function $\eta(t)$, as given in eq S65. **Lower:** The running integral of $\eta(t)$, which plateaus above about 10 ps. The plateau value, as given formally in eq S66, is calculated as the mean in the green shaded region. It is shown as a dotted horizontal line and denoted η_s^t.

Results for SPC/E Water: Figure S10 shows the viscosity spectrum from an NpT simulation of 4096 SPC/E water molecules with force-switching between 1.9 and 2.0 nm for Lennard–Jones forces at 300 K and 1 bar, calculated via eq S64. For pressure coupling, the exponential relaxation barostat was used (`C-rescale` in GROMACS) with time constant `tau-p` of 5 ps. The force fields used, including finite force cut-offs, are the same as for the driven-flow simulations in the main work. The upper panel shows the real and imaginary parts of $\widetilde{\eta}(\nu)$. These are smoothed by convolution with a Gaussian kernel with standard deviation $\sigma = 1.2$ GHz. The resulting steady-state viscosity $\eta_s^\nu = \widetilde{\eta}(\nu = 0)$ is also shown as a horizontal dotted line. The middle panel shows the response function $\eta(t)$, calculated via eq S65. The lower panel shows its running integral. The steady-state viscosity η_s^t, calculated as the average of the data in the plateau region (indicated by the vertical shaded green area), is shown as a horizontal dotted line. The two steady-state viscosities are formally identical, but due to numerical errors, they differ by about 1%. The value reported in the main text, $\eta_{\mathrm{eq}} = 0.698$ mPa s, is the mean of η_s^ν and η_s^t. For all data, error bars are shown as a shaded area above and below the curve, but these are not visible everywhere due to the errors being very small. The errors are calculated by breaking the simulation trajectory into six subtrajectories, and calculating the viscosity independently from each. The mean and error (standard deviation of the mean) can then be extracted for all quantities.

We also carried out the same procedure as shown in Figure S10 on simulation data where the SPC/E-default 0.9 nm Lennard–Jones cutoff was used,[17] and found $\eta_{\mathrm{eq}} = 0.698$ mPa s which agrees exactly with the value for force-switching between 1.9 and 2.0 nm.

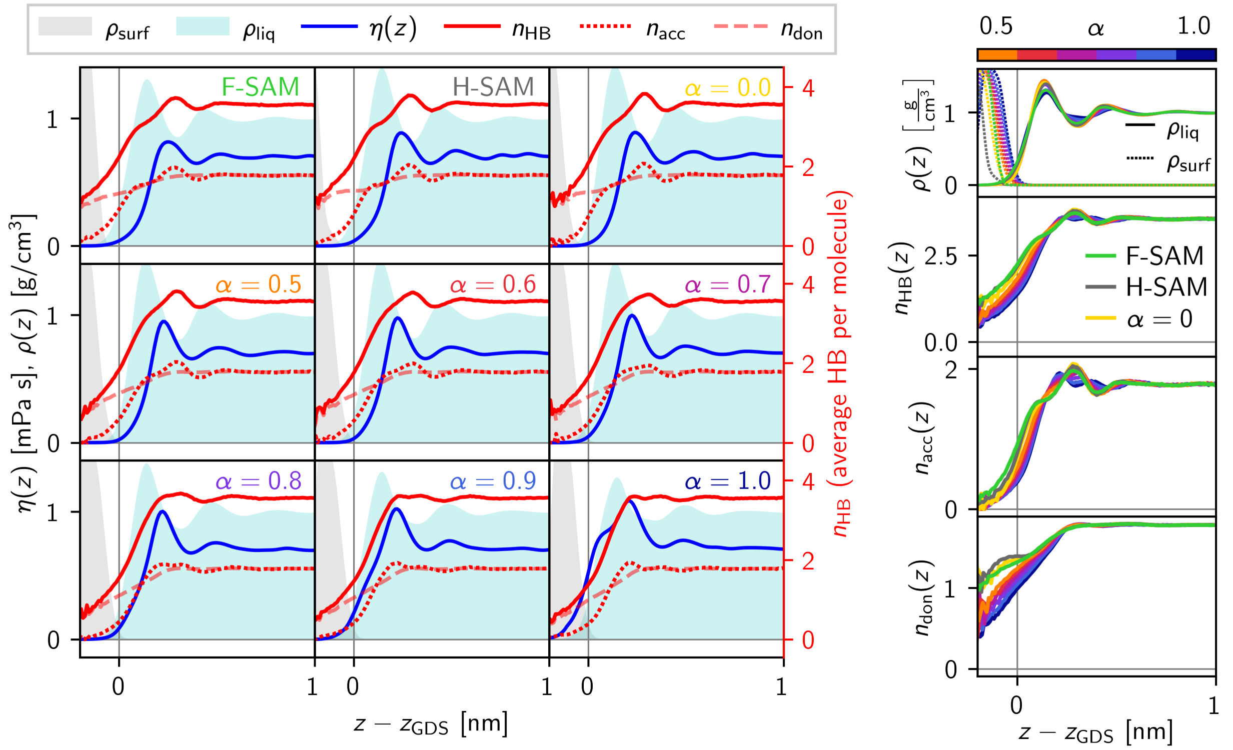

Figure S11: Average number of water-water hydrogen bonds associated with each water molecule, $n_{\mathrm{HB}}(z)$, as a function of molecular center-of-mass z-position, as well as hydrogen bonds *accepted*, $n_{\mathrm{acc}}(z)$, and *donated* per molecule, $n_{\mathrm{don}}(z)$, for all systems studied in this work. **Left:** $n_{\mathrm{HB}}(z)$, $n_{\mathrm{don}}(z)$, and $n_{\mathrm{acc}}(z)$ compared to one another and to the viscosity profile $\eta(z)$ for each system separately. Mass-density profiles of the surface and liquid, $\rho_{\mathrm{surf}}(z)$ and $\rho_{\mathrm{liq}}(z)$, respectively, are shown as shaded areas as a positional reference. **Right:** $n_{\mathrm{HB}}(z)$, $n_{\mathrm{don}}(z)$, and $n_{\mathrm{acc}}(z)$ compared across systems. Here, $\rho_{\mathrm{surf}}(z)$ and $\rho_{\mathrm{liq}}(z)$ are shown in the upper panel.

S14 Hydrogen Bonding Near the Interface

We measure the average number of water-water hydrogen bonds donated n_{don} and accepted n_{acc} per molecule as a function of the z-position of the molecular center of mass. Following Ref. 32, we take two water molecules to be hydrogen bonded if their oxygen–oxygen distance r_{OO} is less than 3.5 Å, and the (donor-hydrogen)–(donor-oxygen)–(acceptor-oxygen) angle, i.e., $\angle \mathrm{HO}_D\mathrm{O}_A$, is less than 30°. Figure S11 plots n_{don}, n_{acc}, and $n_{\mathrm{HB}} = n_{\mathrm{don}} + n_{\mathrm{acc}}$. The left block plots the data system-by-system, comparing it to the viscosity profiles. For the hydrophobic surfaces, the viscosity profiles seem somewhat correlated with n_{acc} and/or n_{HB} across z, that is, the positions of the first and second peaks agree well. This is unsurprising as hydrogen bonding is crucial for water cohesion, and can be expected to play a key role in the viscosity. This helps to explain the bulkward shift of the peak in the viscosity profile relative to the peak in the mass density in the first hydration layer.

The right block compares each profile type across all systems studied in this work, revealing an increase in interfacial water-water hydrogen bonds as surface hydrophobicity increases. This is the opposite trend observed in the main text for the viscosity profiles $\eta(z)$. This is studied more carefully in Figure S12, where the density of water-water hydrogen bonds per unit *volume*, $\rho_{\mathrm{HB}}(z)$, is plotted, again as a function of the z-position of the molecular center of mass. Specifically,

$$\rho_{\mathrm{HB}}(z) = \frac{n_{\mathrm{HB}}(z)\rho_{\mathrm{liq}}(z)}{2m_{\mathrm{mol}}}\,, \qquad \text{(S67)}$$

where $\rho_{\mathrm{liq}}(z)$ is the mass density of water, m_{mol} is the mass of a single water molecule, and the factor 1/2 is included because each hydrogen bond is counted twice in n_{HB}. From $\rho_{\mathrm{HB}}(z)$, the interfacial excess distance of hydrogen bonds, d_{HB}, may also be calculated in the same way as the interfacial viscosity excess distance, d_η, is calculated in the main text, i.e., as the position of the hydrogen-bond dividing surface relative to the Gibbs dividing surface of the water, namely, $z_{\mathrm{HB}} = z_{\mathrm{GDS}} - d_{\mathrm{HB}}$. In Figure S12, $-d_{\mathrm{HB}}$ are plotted as vertical dashed lines. See that d_{HB} is consistently negative, meaning that for all surfaces, there is a deficit of water–water hydrogen bonds at the interface. The inset of Figure S12 plots d_η over d_{HB}, and indeed, the two are negatively correlated across systems. This implies

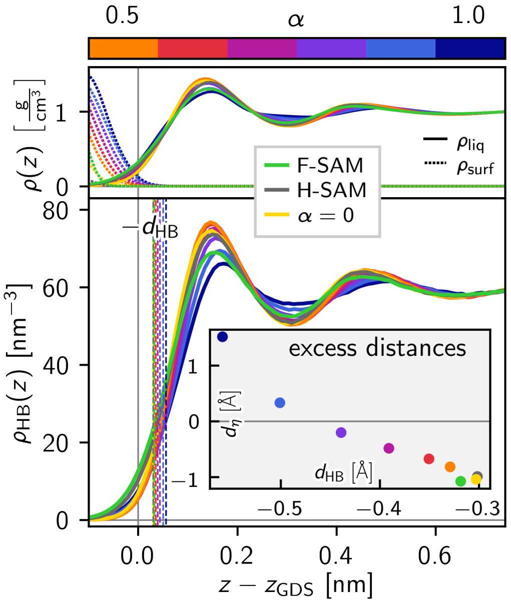

Figure S12: Water-water hydrogen bonds per unit volume, ρ_{HB}, as a function of molecular center-of-mass z-position for all systems studied in this work. Also shown as vertical dashed lines are the corresponding hydrogen-bond dividing surface positions, $z_{\mathrm{HB}} = z_{\mathrm{GDS}} - d_{\mathrm{HB}}$. Mass-density profiles of the surface and liquid, $\rho_{\mathrm{surf}}(z)$ and $\rho_{\mathrm{liq}}(z)$, respectively, are shown in the upper panel as a positional reference. The inset shows the interfacial excess distance of the viscosity, d_η, as a function of d_{HB}.

that the positive excess surface viscosity near more hydrophilic surfaces must result from a different mechanism than an increase in water-water hydrogen bonding there. We hypothesize the mechanism to be the conformational rigidity of water molecules hydrogen-bonded to the surface, which prevents other water molecules from easily flowing past.

S15 Gibbs Dividing Surface

The Gibbs dividing surface defines the position of an interface separating two phases of matter, between which there may in fact be a gradual change in the density and/or composition over some finite distance (usually on the order of angstroms or nanometers). We consider the case of an interface separating a vapor and liquid phase as pictured in Figure S13. The position of the Gibbs dividing surface, z_{GDS}, is taken as the position where the surface excess vanishes, i.e.,

$$z_{\mathrm{GDS}} = z_0 + \int_{z_0}^{z_b} \mathrm{d}z\, \frac{\rho(z_b) - \rho(z)}{\rho(z_b) - \rho(z_0)}\,, \tag{S68}$$

where $\rho(z)$ is the density profile along z, and z_0 and z_b are positions in the vapor and liquid bulk phases, respectively, well away from the interface.[33]

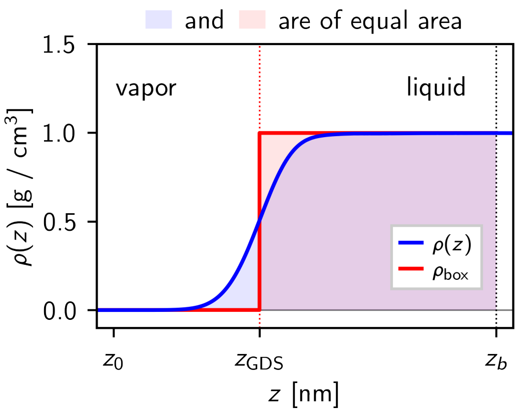

Figure S13: Plot of the density profile of a vapor-liquid water interface meant to illustrate the Gibbs dividing surface. A step-like box profile, with the step positioned at z_{GDS} is also shown. The Gibbs dividing surface is defined such that the red and blue shaded areas are equal, i.e., an integral over $\rho(z)$ from a position z_0 below the liquid, to a position z_b in the bulk, will equal the integral over $\rho_{\mathrm{box}}(z)$ between the same boundaries.

S16 Depletion Length

There is typically a zone of low density at surface–liquid interfaces called the depletion layer, the thickness of which we refer to as the depletion length δ.[34] The depletion length can be quantified by an integral over the relative mass-density deficit. We are interested in the depletion layer specifically as a zone where there is a deficit of occupation by atoms, i.e., where less *space* is taken up on average than in the bulk of the solid or liquid. Thus, we define the depletion length with respect to the packing density ϕ, instead of the usual mass density ρ.[34–36] Here, the packing density ϕ is defined as the unitless ratio of the total van der Waals volume of atoms within a region Ω to the total volume V_Ω of that region, i.e.,

$$\phi(\Omega) = \frac{1}{V_\Omega} \sum_{i\in\Omega} \frac{4}{3}\pi \left(r_i^{\mathrm{vdW}}\right)^3\,, \tag{S69}$$

where r_i^{vdW} is the van der Waals radius of the ith atom in Ω. The van der Waals radii used in this work are experimental values collected in Ref. 37, and are reproduced in Table S2.

atom	C	H	O	F
r^{vdW} [Å]	1.77	1.1	1.58	1.46

Table S2: Experimental van der Waals radii as published in Ref. 37.

For homogeneous phases, such as pure liquids, the mass density and packing density give identical results, but for inhomogeneous phases, such as a SAM with termi-

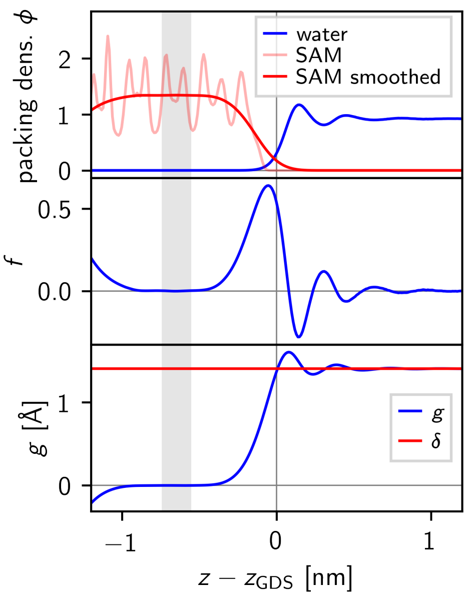

Figure S14: Plots illustrating the extraction of the depletion length for the F-SAM/water system. The upper panel shows packing density profiles for the SAM and water, ϕ_s and ϕ_l, respectively. The density profile ϕ_s of the SAM is smoothed by Gaussian convolution, which preserves the integral, in order to find the bulk density, which is calculated as the mean in the gray shaded region. The middle and lower panels show f and g (see eqs S70 and S71, respectively). The red horizontal line in the lower panel is the resulting depletion length δ.

nal OH-groups, they give different results. Thus, the depletion length is defined as

$$\delta = \int_{z_s}^{z_l} \mathrm{d}z\, f(z) = \int_{z_s}^{z_l} \mathrm{d}z \left(1 - \frac{\phi_s(z)}{\phi_s^b} - \frac{\phi_l(z)}{\phi_l^b}\right), \tag{S70}$$

where $\phi_s(z)$ and $\phi_l(z)$ are the surface and liquid packing density profiles, ϕ_s^b and ϕ_l^b are their respective bulk values, and z_s and z_l are z-positions well within the bulk of the surface and liquid respectively.[34–36,38,39] The integrand $f(z)$ is the density deficit and its running integral we define as

$$g(z) = \int_{z_s}^{z} \mathrm{d}z'\, f(z'). \tag{S71}$$

The upper panel of Figure S14 shows plots of packing densities of liquid water and an F-SAM. The density of the SAM is smoothed by convolution with a Gaussian in order to facilitate determination of ϕ_s^b, which preserves the integral. The middle and lower panels show the corresponding $f(z)$ and $g(z)$. The mean value for the SAM density ϕ_s^b is taken over the shaded region in the plot. The depletion length δ may be calculated from the difference in the bulk values of $g(z)$ in the solid and liquid, as indicated in the figure.

S17 The Friction–Wettability Relationship

The motion of an interfacial liquid molecule tangential to a surface can be treated as a series of barrier crossings over a corrugated potential landscape. Take the potential landscape $U(x)$ to be a periodic potential with a minimum at $x = 0$, amplitude U_0, and $2L$-periodicity. A particle in a well has to cross a barrier of height U_0 to move one well to the left or right. From transition state theory, the mean barrier crossing time τ over a barrier of height U_0 is

$$\tau = \tau_0 e^{\beta U_0}, \tag{S72}$$

where $\beta = (k_B T)^{-1}$ is the inverse thermal energy and τ_0 is a characteristic time.[40,41] Consider the case of a small, constant force F applied to the particle ($LF \ll U_0$, $LF \ll \beta^{-1}$). This changes the barrier heights on the left and right of the particle to $U_l \approx U_0 + LF$ and $U_r \approx U_0 - LF$. Letting the average rate of barrier crossings to the left and right be given by τ_l and τ_r, the average velocity of the particle is

$$\begin{aligned} v &= 2L\left(\frac{1}{\tau_r} - \frac{1}{\tau_l}\right) \\ &= \frac{2L}{\tau_0}\left(e^{-\beta U_r} - e^{-\beta U_l}\right) \\ &\approx \frac{2L}{\tau_0}\left(e^{-\beta(U_0 - LF)} - e^{-\beta(U_0+LF)}\right) \\ &= \frac{2L}{\tau_0} e^{-\beta U_0}\left(e^{\beta LF} - e^{-\beta LF}\right) \\ &= \frac{4L}{\tau_0} e^{-\beta U_0} \sinh(\beta LF) \\ &\approx \frac{4L}{\tau_0} e^{-\beta U_0} \beta LF, \end{aligned} \tag{S73}$$

where in the final step it is used that $\sinh x \approx x$ for small x up to third order. Let there be n interfacial particles per unit area, then the stress driving the velocity v is $-F_f = nF$. Substituting and rearranging gives

$$F_f = -\frac{n\tau_0 e^{\beta U_0}}{4\beta L^2} v. \tag{S74}$$

Taking the velocity v to be the slip velocity u_{slip} gives for the Navier friction coefficient

$$\lambda = \frac{n\tau_0 e^{\beta U_0}}{4\beta L^2} \propto e^{\beta U_0}. \tag{S75}$$

Here it is assumed that n is constant in U_0, which we justify by saying that all liquid molecules directly adjacent to the surface see the corrugated potential landscape, and this number is relatively constant over the different systems studied in this work. The potential landscape $U(x)$ seen by the particle is in fact the sum of all surface–liquid interactions along its tangential trajectory. Therefore, it should hold that $U_0 \propto \varepsilon_{\text{int}}$, where

$\varepsilon_{\mathrm{int}}$ is the areal surface–liquid interaction energy. The Young-Dupré equation relates the areal work of adhesion W to the wetting coefficient $k = \cos\theta$ (where θ is the contact angle) and liquid–vapor interfacial tension γ_{lv},

$$W = \gamma_{lv}\,(k+1)\;. \tag{S76}$$

In the approximation $W \propto \varepsilon_{\mathrm{int}}$ then,

$$U_0 \propto k+1\,, \tag{S77}$$

and eqs S75 and S76 give a scaling relation between the steady-state Navier friction coefficient λ and the contact angle

$$\lambda \propto e^{A(k+1)}\,, \tag{S78}$$

where A is a positive numerical constant. In Ref. 42, scaling relationships are derived starting from the Fokker-Planck equation and assuming the potential landscape has the form

$$U(x) = -U_0 \frac{1-\cos\left(\frac{\pi x}{L}\right)}{2}\,. \tag{S79}$$

The result for the friction coefficient is

$$\lambda \propto \int_0^{2L} \mathrm{d}x\, e^{\beta U\left(\frac{x}{2L}\right)} \int_0^{2L} \mathrm{d}x\, e^{-\beta U\left(\frac{x}{2L}\right)}\,. \tag{S80}$$

In the high-barrier limit ($U_0 \to \infty$) this gives

$$\lambda \propto \frac{e^{\beta U_0}}{U_0}\,. \tag{S81}$$

In the low-barrier limit ($U_0 \to 0$) it gives, to third order,

$$\lambda \propto 1 + \frac{(\beta U_0)^2}{16}\,. \tag{S82}$$

This quadratic relationship has also been explored in other publications.[36,43]

Figure S15 compares fits of eqs S78, S81 and S82 to the friction-wetting data for all systems studied in this work. In the upper panel of Figure S15, which has a linear y-axis, the data are fit directly with the fitting functions. In the lower panel, which has a logarithmic y-axis, the fit parameters are obtained in logarithmic space, i.e., by fitting the logarithm of the y-data with the logarithm of the respective fitting function. The resulting fitted functions are printed directly in the corresponding panels. Table S3 shows the sum of squared residuals of the fits. The exponential fits (eqs S78 and S81) seem to capture the behavior much more accurately than the quadratic fit, as is also apparent from Table S3. This is perhaps to be expected as the low-barrier limit is a poor approximation for hydrophilic surfaces like the (α=0.8)- and (α=0.9)-SAMs. The two exponential fits, (eqs S78 and S81) seem to fit the data with a roughly similar degree of accuracy, with Table S3 indicating that the fit of eq S81 is marginally more accurate. As the fits are of similar quality, we reproduce the fit of eq S78 in Figure 3 in the main text, as the simple exponential function better facilitates comparison with the depletion length and viscosity excess distance.

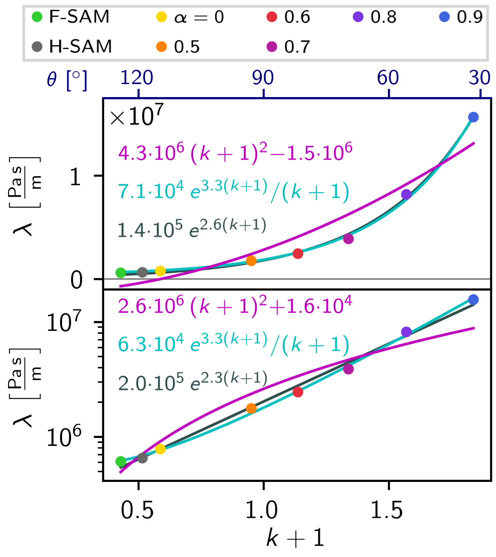

Figure S15: A comparison of exponential (eqs S78 and S81) and quadratic (eq S82) fits of the Navier friction coefficient λ over $k+1$, where $k = \cos\theta$ is the wetting coefficient, for all systems studied in this work, aside from the (α=1)-SAM/water system. While the data plotted in the two panels are the same, the scale of the y-axis is linear for the upper panel and logarithmic for the lower panel, and the fits in the lower panel are performed in logarithmic space.

	fit of y (upper)	fit of $\log y$ (lower)
eq S78: $Ae^{B(k+1)}$	7.134×10^{10}	1.106×10^{-2}
eq S81: $Ae^{B(k+1)}/(k+1)$	6.902×10^{10}	3.724×10^{-3}
eq S82: $A(k+1)^2+B$	3.248×10^{12}	1.144×10^{-1}

Table S3: The sum of squared residuals for the fits shown in Figure S15. For the right column, the residuals are calculated in logarithmic space.

References

(1) Strong, L.; Whitesides, G. M. Structures of self-assembled monolayer films of organosulfur compounds adsorbed on gold single crystals: electron diffraction studies. *Langmuir* **1988**, *4*, 546–558.

(2) Chidsey, C. E. D.; Loiacono, D. N. Chemical functionality in self-assembled monolayers: structural and electrochemical properties. *Langmuir* **1990**, *6*, 682–691.

(3) Ulman, A.; Eilers, J. E.; Tillman, N. Packing and molecular orientation of alkanethiol monolayers on gold surfaces. *Langmuir* **1989**, *5*, 1147–1152.

(4) Alves, C. A.; Porter, M. D. Atomic force microscopic characterization of a fluorinated alkanethiolate monolayer at gold

and correlations to electrochemical and infrared reflection spectroscopic structural descriptions. *Langmuir* **1993**, *9*, 3507–3512.

(5) Liu, G.; Fenter, P.; Chidsey, C. E. D.; Ogletree, D. F.; Eisenberger, P.; Salmeron, M. An unexpected packing of fluorinated n-alkane thiols on Au(111): A combined atomic force microscopy and x-ray diffraction study. *J. Chem. Phys.* **1994**, *101*, 4301–4306.

(6) Jaschke, M.; Schönherr, H.; Wolf, H.; Butt, H.-J.; Bamberg, E.; Besocke, M. K.; Ringsdorf, H. Structure of Alkyl and Perfluoroalkyl Disulfide and Azobenzenethiol Monolayers on Gold(111) Revealed by Atomic Force Microscopy. *J. Phys. Chem.* **1996**, *100*, 2290–2301.

(7) Carlson, S.; Becker, M.; Brünig, F. N.; Ataka, K.; Cruz, R.; Yu, L.; Tang, P.; Kanduč, M.; Haag, R.; Heberle, J.; Makki, H.; Netz, R. R. Hydrophobicity of Self-Assembled Monolayers of Alkanes: Fluorination, Density, Roughness, and Lennard-Jones Cutoffs. *Langmuir* **2021**, *37*, 13846–13858.

(8) Berendsen, H.; van der Spoel, D.; van Drunen, R. GROMACS: A message-passing parallel molecular dynamics implementation. *Comput. Phys. Commun.* **1995**, *91*, 43–56.

(9) Lindahl, E.; Hess, B.; Van Der Spoel, D. GROMACS 3.0: a package for molecular simulation and trajectory analysis. *Mol. model. ann.* **2001**, *7*, 306–317.

(10) Hockney, R.; Goel, S.; Eastwood, J. Quiet high-resolution computer models of a plasma. *J. Comput. Phys.* **1974**, *14*, 148–158.

(11) Bussi, G.; Donadio, D.; Parrinello, M. Canonical sampling through velocity rescaling. *J. Chem. Phys.* **2007**, *126*, 014101.

(12) Carlson, S. R.; Schullian, O.; Becker, M. R.; Netz, R. R. Modeling Water Interactions with Graphene and Graphite via Force Fields Consistent with Experimental Contact Angles. *J. Phys. Chem. Lett.* **2024**, *15*, 6325–6333.

(13) Darden, T.; York, D.; Pedersen, L. Particle mesh Ewald: An $N \cdot \log(N)$ method for Ewald sums in large systems. *J. Chem. Phys.* **1993**, *98*, 10089–10092.

(14) Jorgensen, W. L.; Madura, J. D.; Swenson, C. J. Optimized intermolecular potential functions for liquid hydrocarbons. *J. Am. Chem. Soc.* **1984**, *106*, 6638–6646.

(15) Kaminski, G.; Duffy, E. M.; Matsui, T.; Jorgensen, W. L. Free Energies of Hydration and Pure Liquid Properties of Hydrocarbons from the OPLS All-Atom Model. *J. Phys. Chem.* **1994**, *98*, 13077–13082.

(16) Jorgensen, W. L.; Maxwell, D. S.; Tirado-Rives, J. Development and Testing of the OPLS All-Atom Force Field on Conformational Energetics and Properties of Organic Liquids. *J. Am. Chem. Soc.* **1996**, *118*, 11225–11236.

(17) Berendsen, H. J. C.; Grigera, J. R.; Straatsma, T. P. The missing term in effective pair potentials. *J. Phys. Chem.* **1987**, *91*, 6269–6271.

(18) Vargaftik, N. B.; Volkov, B. N.; Voljak, L. D. International Tables of the Surface Tension of Water. *J. Phys. Chem. Ref. Data* **1983**, *12*, 817–820.

(19) Navier, C. *Mémoire de l'Académie des sciences de l'Institut de France*; Gauthier-Villars: Paris, 1823; Vol. 6; pp 389–440.

(20) Kiefer, H.; Vitali, D.; Dalton, B. A.; Scalfi, L.; Netz, R. R. Effect of frequency-dependent shear and volume viscosities on molecular friction in liquids. *Phys. Rev. E* **2025**, *111*, 015104.

(21) Todd, B. D.; Hansen, J. S.; Daivis, P. J. Nonlocal Shear Stress for Homogeneous Fluids. *Phys. Rev. Lett.* **2008**, *100*, 195901.

(22) Stokes, G. G. On the Theories of the Internal Friction of Fluids in Motion, and of the Equilibrium and Motion of Elastic Solids. *Trans. Cambridge Philos. Soc.* **1845**, *8*, 287–341.

(23) Stokes, G. G. On the Effect of the Internal Friction of Fluids on the Motion of Pendulums. *Trans. Cambridge Philos. Soc.* **1851**, *9*, 8.

(24) Kanduč, M.; Eixeres, L.; Liese, S.; Netz, R. R. Generalized line tension of water nanodroplets. *Phys. Rev. E* **2018**, *98*, 032804.

(25) Bonthuis, D. J.; Netz, R. R. Beyond the Continuum: How Molecular Solvent Structure Affects Electrostatics and Hydrodynamics at Solid–Electrolyte Interfaces. *J. Phys. Chem. B* **2013**, *117*, 11397–11413.

(26) Schlaich, A.; Kappler, J.; Netz, R. R. Hydration Friction in Nanoconfinement: From Bulk via Interfacial to Dry Friction. *Nano Lett.* **2017**, *17*, 5969–5976.

(27) Wolde-Kidan, A.; Netz, R. R. Interplay of Interfacial Viscosity, Specific-Ion, and Impurity Adsorption Determines Zeta Potentials of Phospholipid Membranes. *Langmuir* **2021**, *37*, 8463–8473.

(28) González, M. A.; Abascal, J. L. F. The shear viscosity of rigid water models. *J. Chem. Phys.* **2010**, *132*, 096101.

(29) Schulz, J. C. F.; Schlaich, A.; Heyden, M.; Netz, R. R.; Kappler, J. Molecular interpretation of the non-Newtonian viscoelastic behavior of liquid water at high frequencies. *Phys. Rev. Fluids* **2020**, *5*, 103301.

(30) Alfè, D.; Gillan, M. J. First-Principles Calculation of Transport Coefficients. *Phys. Rev. Lett.* **1998**, *81*, 5161–5164.

(31) Daivis, P. J.; Evans, D. J. Comparison of constant pressure and constant volume nonequilibrium simulations of sheared model decane. *J. Chem. Phys.* **1994**, *100*, 541–547.

(32) Luzar, A.; Chandler, D. Effect of Environment on Hydrogen Bond Dynamics in Liquid Water. *Phys. Rev. Lett.* **1996**, *76*, 928–931.

(33) Bonthuis, D. J.; Gekle, S.; Netz, R. R. Profile of the Static Permittivity Tensor of Water at Interfaces: Consequences for Capacitance, Hydration Interaction and Ion Adsorption. *Langmuir* **2012**, *28*, 7679–7694.

(34) Maccarini, M.; Steitz, R.; Himmelhaus, M.; Fick, J.; Tatur, S.; Wolff, M.; Grunze, M.; Janeček, J.; Netz, R. R. Density Depletion at Solid–Liquid Interfaces: a Neutron Reflectivity Study. *Langmuir* **2007**, *23*, 598–608.

(35) Sedlmeier, F.; Janeček, J.; Sendner, C.; Bocquet, L.; Netz, R. R.; Horinek, D. Water at polar and nonpolar solid walls (Review). *Biointerphases* **2008**, *3*, FC23–FC39.

(36) Sendner, C.; Horinek, D.; Bocquet, L.; Netz, R. R. Interfacial Water at Hydrophobic and Hydrophilic Surfaces: Slip, Viscosity, and Diffusion. *Langmuir* **2009**, *25*, 10768–10781.

(37) Rowland, R. S.; Taylor, R. Intermolecular Nonbonded Contact Distances in Organic Crystal Structures: Comparison with Distances Expected from van der Waals Radii. *J. Phys. Chem.* **1996**, *100*, 7384–7391.

(38) Mamatkulov, S. I.; Khabibullaev, P. K.; Netz, R. R. Water at Hydrophobic Substrates: Curvature, Pressure, and Temperature Effects. *Langmuir* **2004**, *20*, 4756–4763.

(39) Janeček, J.; Netz, R. R. Interfacial Water at Hydrophobic and Hydrophilic Surfaces: Depletion versus Adsorption. *Langmuir* **2007**, *23*, 8417–8429.

(40) Kramers, H. A. Brownian motion in a field of force and the diffusion model of chemical reactions. *Physica* **1940**, *7*, 284–304.

(41) Brünig, F. N.; Netz, R. R.; Kappler, J. Barrier-crossing times for different non-Markovian friction in well and barrier: A numerical study. *Phys. Rev. E* **2022**, *106*, 044133.

(42) Erbaş, A.; Horinek, D.; Netz, R. R. Viscous Friction of Hydrogen-Bonded Matter. *J. Am. Chem. Soc.* **2012**, *134*, 623–630.

(43) Huang, D. M.; Sendner, C.; Horinek, D.; Netz, R. R.; Bocquet, L. Water Slippage versus Contact Angle: A Quasiuniversal Relationship. *Phys. Rev. Lett.* **2008**, *101*, 226101.
